# Induced PTF1a expression in pancreatic ductal adenocarcinoma cells activates acinar gene networks, reduces tumorigenic properties, and sensitizes cells to gemcitabine treatment

**DOI:** 10.1002/1878-0261.12314

**Published:** 2018-05-21

**Authors:** Brad L. Jakubison, Patrick G. Schweickert, Sarah E. Moser, Yi Yang, Hongyu Gao, Kathleen Scully, Pamela Itkin‐Ansari, Yunlong Liu, Stephen F. Konieczny

**Affiliations:** ^1^ Department of Biological Sciences Purdue University West Lafayette IN USA; ^2^ Bindley Bioscience Center Purdue University West Lafayette IN USA; ^3^ Purdue University Center for Cancer Research Purdue University West Lafayette IN USA; ^4^ Laboratory for Computational Genomics Indiana University School of Medicine Indianapolis IN USA; ^5^ Development and Aging Program Sanford‐Burnham Medical Research Institute La Jolla CA USA

**Keywords:** ABC transporters, bHLH, exocrine pancreas, MIST1, PDAC, transcription

## Abstract

Pancreatic acinar cells synthesize, package, and secrete digestive enzymes into the duodenum to aid in nutrient absorption and meet metabolic demands. When exposed to cellular stresses and insults, acinar cells undergo a dedifferentiation process termed acinar–ductal metaplasia (ADM). ADM lesions with oncogenic mutations eventually give rise to pancreatic ductal adenocarcinoma (PDAC). In healthy pancreata, the basic helix‐loop‐helix (bHLH) factors MIST1 and PTF1a coordinate an acinar‐specific transcription network that maintains the highly developed differentiation status of the cells, protecting the pancreas from undergoing a transformative process. However, when *MIST1* and *PTF1a* gene expression is silenced, cells are more prone to progress to PDAC. In this study, we tested whether induced *MIST1* or *PTF1a* expression in PDAC cells could (i) re‐establish the transcriptional program of differentiated acinar cells and (ii) simultaneously reduce tumor cell properties. As predicted, PTF1a induced gene expression of digestive enzymes and acinar‐specific transcription factors, while MIST1 induced gene expression of vesicle trafficking molecules as well as activation of unfolded protein response components, all of which are essential to handle the high protein production load that is characteristic of acinar cells. Importantly, induction of PTF1a in PDAC also influenced cancer‐associated properties, leading to a decrease in cell proliferation, cancer stem cell numbers, and repression of key ATP‐binding cassette efflux transporters resulting in heightened sensitivity to gemcitabine. Thus, activation of pancreatic bHLH transcription factors rescues the acinar gene program and decreases tumorigenic properties in pancreatic cancer cells, offering unique opportunities to develop novel therapeutic intervention strategies for this deadly disease.

**Abbreviations**
ABC: ATP‐binding cassette
ADM: acinar–ductal metaplasia
bHLH: basic helix‐loop‐helix
CSC: cancer stem cell
DEG: differentially expressed gene
Dox: doxycycline
GSEA: Gene Set Enrichment Analysis
IC_50_: half‐maximal inhibitory concentration
IHC: immunohistochemistry
IPA: ingenuity pathway analysis
KEGG: Kyoto encyclopedia of genes and genomes
KPC: Cre recombinase‐driven LSL‐Kras^G12D^; LSL‐Trp53^R172H^ mouse model
PanIN: pancreatic intraepithelial neoplasia
PDAC: pancreatic ductal adenocarcinoma
UPR: unfolded protein response

## 1 Introduction

Pancreatic ductal adenocarcinoma (PDAC) is a dismal disease with a 5‐year survival rate of 8% (American Cancer Society, 2017). Even more alarming is the minimal progress that has been made to improve patient outcome over the past 30 years. Current efforts to treat this disease revolve around the use of gemcitabine – a nucleoside analog; FOLFIRINOX – a combinatorial drug regimen; and erlotinib – a tyrosine kinase inhibitor of the epidermal growth factor receptor (Conroy *et al*., 2011; Moore *et al*., 2007; Réjiba *et al*., 2009). Unfortunately, these and other agents have produced only marginal results in increasing life expectancy (American Cancer Society, 2017; Heinemann *et al*., 2000). Combined with issues regarding the availability of early diagnostic tools, it is clear that alternative strategies that look toward comprehensive networks are needed to more efficiently treat patients.

The formation of pancreatic tumors requires a sequence of oncogenic and tumor suppressor insults to drive healthy acinar cells toward a proliferative, dysregulated tumor cell phenotype (Bryant *et al*., 2014; Hruban *et al*., 2000; Pasca *et al*., 2006; Witkiewicz *et al*., 2015). Oncogenic *KRAS* is thought to be the primary driver of PDAC and readily transforms cells that have undergone acinar–ductal metaplasia (ADM), resulting in a dedifferentiated state where the proacinar basic helix‐loop‐helix (bHLH) transcription factor genes *MIST1* and *PTF1a* are transcriptionally silenced (Adell *et al*., 2000; Day *et al*., 1996; Krah *et al*., 2015; Rodolosse *et al*., 2004; Guanglu Shi *et al*., 2009; Zhu *et al*., 2007). In healthy pancreata, MIST1 facilitates cell‐to‐cell communication, promotes acinar cell polarity, controls Ca^2+^ flux, promotes unfolded protein response (UPR) homeostasis, aids in vesicle assembly, and is required for regulated exocytosis (Direnzo *et al*., 2012; Garside *et al*., 2010; Hess *et al*., 2016; Jia *et al*., 2008; Rukstalis *et al*., 2003). PTF1a is necessary for pancreatic organogenesis and maintenance of gene expression networks associated with the production of a vast array of digestive hydrolases (Beres *et al*., 2006; Direnzo *et al*., 2012; Garside *et al*., 2010; Hess *et al*., 2016; Jia, 2008; Jiang *et al*., 2016; Masui *et al*., 2007). Importantly, both MIST1 and PTF1a negatively regulate cell cycle progression by inducing *p21* ^*CIP1/WAF1*^ expression (Jia *et al*., 2008; Rodolosse *et al*., 2004).

Silencing of the *MIST1* or *PTF1a* genes results in significant changes to acinar cells, leading to widespread failure to appropriately synthesize and secrete digestive enzymes, maintain proper apical–basal polarity, and retain essential gap junctions that permit intercellular communication (Direnzo *et al*., 2012; Jiang *et al*., 2016; Krah *et al*., 2015; Murtaugh and Leach, 2007). In normal pancreata, the silencing of *MIST1* and *PTF1a* during injury permits transient acinar cell regeneration, allowing the exocrine organ to recover from damage (Karki *et al*., 2015; Krah *et al*., 2015). However, in conditions where oncogenic insults have accrued, the process promotes tumorigenesis. Importantly, loss of these transcription factors is instrumental in the progression of pancreatic disease states. *MIST1*‐null (*MIST1* ^*KO*^) acinar cells with activated *Kras* mutations greatly accelerate the formation of precancerous pancreatic intraepithelial neoplasia (PanIN) lesions (Shi *et al*., 2009, 2013). Conversely, forced expression of MIST1 prevents this increased rate of KRAS‐induced transformation (Shi *et al*., 2013). Additionally, recent work has shown that disruption of the bHLH:HLH axis in PDAC cells through forced expression of E47 leads to accumulation of MIST1, decreased cellular proliferation, and reduction in tumor formation (Kim *et al*., 2015). Similar results have been observed with PTF1a where loss of PTF1a potentiates the development of ADM and PanIN lesions as the cells no longer maintain expression of the acinar gene program (Campos *et al*., 2013; Kondratyeva *et al*., 2017; Krah *et al*., 2015). Thus, MIST1 and PTF1a protect acinar cells against the development of the earliest stages of pancreatic cancer.

Given the importance of bHLH factors to normal cellular function, we hypothesized that induced expression of the acinar‐specific bHLH differentiation factors MIST1 and PTF1a in PDAC cells would result in stimulation of the acinar gene network and decreased tumorigenic potential. Indeed, doxycycline‐inducible MIST1 and PTF1a retained transcriptional activity in PDAC cells. MIST1 induced expression of the vesicle trafficking genes *RAB26* and *RAB3D* as well as genes associated with the UPR, whereas PTF1a induced key acinar transcription factors and an array of digestive enzyme genes. Forced expression of PTF1a also resulted in decreased tumor‐associated gene expression profiles which led to decreased cell proliferation, decreased pancreatic cancer stem cells (CSCs), and a significant increase in sensitivity toward gemcitabine treatment. Together, these studies promote the concept that strategies to induce an acinar differentiation program in PDAC tumor cells may have high efficacy in reversing the aggressive nature of this disease.

## 2 Materials and methods

### 2.1 Plasmid constructs

The open reading frames of mouse PTF1a^myc^ and rat MIST1^myc^ were cloned into the Tet‐One™ plasmid (Clontech Laboratories, Inc., Mountain View, CA, USA) by standard procedures. Pgl3 RBPJ‐L (gift from Raymond McDonald) and TA‐E‐Box‐Luc reporters have been previously described (Masui *et al*., 2007; Tran *et al*., 2007).

### 2.2 Cell culture

The KC mouse line was generated from *elastase‐CreER, LSL‐Kras* ^*G12D/+*^ PDAC tumors, while KPC1 and KPC2 lines were generated from *elastase‐CreER, LSL‐Kras* ^*G12D/+*^ *, Tpr53* ^*R172H/+*^ PDAC tumors (Y. Yang & S. F. Konieczny, unpublished data). KC, KPC1, KPC2, and Panc‐1 cells (ATCC) were cultured in high‐glucose Dulbecco's modified Eagle's medium (DMEM) supplemented with 10% FBS, 1% penicillin/streptomycin at 5% CO_2_, 37 °C. Cells were transfected with the empty Tet‐One, Tet‐PTF1a^myc^, and Tet‐MIST1^myc^ plasmids using X‐tremeGENE 9 (Cat. 06365787001, Roche, Indianapolis, IN, USA), and stable transformants were selected for growth in 3.0 μg·mL^−1^ puromycin for a period of two weeks. Individual Panc‐1 Tet‐One, Panc‐1 Tet‐PTF1a, and Panc‐1 Tet‐MIST1 clones were screened for appropriate doxycycline induction of PTF1a and MIST1 expression, respectively, using 1 μg·mL^−1^ doxycycline hyclate (Cat. D3447, Sigma, St. Louis, MO, USA) for a period of 72 h unless otherwise stated. Doxycycline was replaced every 48 h along with fresh media. All cell lines were genetically authenticated by the American Type Culture Collection and pathogen‐tested by IDEXX Laboratories.

### 2.3 RNA‐Seq analysis

Four biological replicates of Panc‐1 Tet‐MIST1, Tet‐PTF1a, and control Tet‐One cells were incubated with or without 1 μg·mL^−1^ doxycycline for a period of 72 h, followed by RNA isolation using the Qiagen miRNeasy extraction kit (Cat. 217004, Qiagen, Hilden, Germany). Illumina HiSeq 4000 sequencing was used to generate 50M paired‐end reads per sample, and reads were aligned to human reference genome hg19 using TopHat. A filter of > 0.5 counts per million reads (roughly equivalent to 10 reads) in at least four samples was implemented prior to determining gene expression using edgeR (Robinson *et al*., 2010). Genes with an FDR < 0.05 were considered differentially expressed with no additional filter for fold change. Differentially expressed genes (DEGs) were analyzed using functional enrichment analysis with ingenuity pathway analysis software (Ingenuity Systems, Inc., Redwood City, CA, USA) and Gene Set Enrichment Analysis (GSEA, Broad Institute, Cambridge, MA, USA). The GSEA pancreatic CSC marker list was curated using published pancreatic stem cell markers (Dalla Pozza *et al*., 2015; Kure *et al*., 2012; Li *et al*., 2007). Kyoto encyclopedia of genes and genomes (KEGG) pathway analysis (Kanehisa and Goto, 2000) was also performed independently on the induced and repressed DEGs.

AME from meme suite v4.12.0 was used to determine the enrichment of PTF1a and MIST1 motifs within the promoter regions of DEGs (McLeay and Bailey, 2010). Promoter regions were defined as −1000 to +100 base pairs from the TSS. Promoter sequences were retrieved from genome build hg19 using BEDTools v.2.27.1 (Quinlan and Hall, 2010). PTF1a motifs containing an E box and TC box separated by either one or two helical turns midpoint to midpoint, as described previously, were used as input motifs (Cockell *et al*., 1989; Rose and MacDonald, 1997; Thompson *et al*., 2012) for analysis of the Tet‐PTF1a dataset. GC and TC E box motifs were used as motif inputs for analysis of the Tet‐MIST1 dataset. Additionally, *de novo* motif discovery was performed using homer v4.8 (Salk Institute, San Diego, CA, USA) (Heinz *et al*., 2010). For all motif analyses, induced and repressed genes were analyzed independently.

For comparison of the RNA‐Seq results to published ChIP‐Seqs of PTF1a (https://www.ncbi.nlm.nih.gov/geo/query/acc.cgi?acc=GSE86262) and MIST1 (https://www.ncbi.nlm.nih.gov/geo/query/acc.cgi?acc=GSE86289), previously called peaks were first downloaded from GEO (http://www.ncbi.nlm.nih.gov/geo/). great v3.0.0 software (McLean *et al*., 2010) was then used, with a limitation on distal associations of up to 20 kb, to determine what genes were associated with ChIP‐Seq peaks. Because the ChIP‐Seq experiments were performed on mouse samples while the RNA‐Seqs were generated from human cells, genes with ChIP‐Seq peaks were first converted to human orthologs using Ensembl BioMart and then examined to determine overlaps between datasets.

### 2.4 Immunofluorescence microscopy

Pancreatic tissue sections were processed as previously described (Karki *et al*., 2015). Panc‐1 cells were fixed in 10% neutral buffered formalin, washed with PBS, and permeabilized with 0.3% Triton X‐100 in PBS. Cells were incubated for 1 h at RT with the following primary antibodies: CPA1 (1 : 100, cat. 131901; BD Biosciences, San Jose, CA, USA), PRSS2 (1:100, cat. sab1400226; Sigma), MIST1 (1 : 500, in‐house affinity‐purified antibody), MYC (1 : 500, cat. 9E10; Developmental Hybridoma Bank, Iowa City, IA, USA), and PTF1a (1 : 2000, gift from Chris Wright). Samples were incubated for 1 h with a 1 : 200 dilution of goat anti‐mouse or goat anti‐rabbit secondary antibody conjugated to biotin followed by a 5‐min incubation with Avidin–Alexa Fluor 488 (Cat. S11223, Invitrogen, Carlsbad, CA, USA) or Avidin–Alexa Fluor 594 (Cat. S11227, Invitrogen). Nuclei were counterstained using Vectashield hard mount with DAPI (Cat. H‐1500, Vector Labs, Burlingame, CA, USA). A full listing of antibodies used in this study is provided in Table S1.

### 2.5 Real‐time qPCR analysis

Total RNA was extracted using the RNeasy Mini Kit (Cat. 217004, Qiagen). cDNA was synthesized (1 μg per sample) using the iScript cDNA synthesis kit (Cat. 1708891, Bio‐Rad, Hercules, CA, USA). Quantitative RT‐PCR was performed on a Roche LightCycler 96 System with SYBR Green (Cat. 04913850001, Roche). Human pancreas RNA used for RT‐qPCR analysis was obtained from Life Technologies (Cat. AM7954, lot 1908572, Carlsbad, CA, USA). All amplicons were sequenced at the Purdue University Sequencing Core to verify correct targets. Gene expression was normalized to 18S rRNA. Primer sequences are provided in Table S2. Three to six biological replicates were performed with two technical replicates for each assay. Data were analyzed using the delta‐delta CT method to determine fold change differences.

### 2.6 Flow cytometry

Panc‐1 Tet‐One, Tet‐MIST1, and Tet‐PTF1a cells were dosed with 1 μg·mL^−1^ doxycycline and incubated at 5% CO_2_, 37 °C. The medium was changed after 48 h, and cells were harvested at 72 h. Cells were pelleted, washed, and incubated with blocking antibody CD16/32 (Cat. 2.4G2, BD Pharmingen, Franklin Lakes, NJ, USA), washed twice with PBS, and then incubated with FITC‐conjugated mouse anti‐human CD24 (1 : 100, Cat. 560992; BD Biosciences) and Cy5‐conjugated CD44 (1 : 100, Cat. 553135; BD Biosciences) for 30 min at 4 °C. Cells were analyzed for coexpression of CD24 and CD44 antigens using the BD Fortessa Cell Analyzer in the Purdue University Bindley Biosciences Flow Cytometry Core. Gates were set using a no‐antibody control. Assays were performed with a minimum of three biological replicates.

### 2.7 Immunoblotting

Cells were treated for 72 h with 1 μg·mL^−1^ doxycycline unless otherwise stated. Protein lysates were harvested using standard RIPA buffer containing sodium vanadate (Cat. 450253; Sigma) as well as protease inhibitors (Cat. COEDTAF‐RO; Roche) and phosphatase inhibitors (Cat. P5726 and P0044; Roche) and sonicated using a Vibra Cell Sonicator. Wild‐type pancreata and tumor tissues were harvested in RIPA as above, homogenized using a Tissue Tearor (model 985370) and sonicated using a Vibra Cell Sonicator. Membranes were incubated for 1 h at room temperature with primary antibodies to Myc (1 : 500, Cat. 9E10; Developmental Hybridoma Bank), α/β HSP90 (1 : 5000, Cat. SC‐7947; Santa Cruz, Santa Cruz, CA, USA), MIST1 (1 : 1000, in‐house affinity‐purified antibody), PTF1a (1 : 4000, gift from Chris Wright), and SOX9 (1 : 3000, Cat. ab5535; Millipore, Burlington, MA, USA) for 1 h. Immunoblots were washed and blotted with HRP‐conjugated anti‐mouse and anti‐rabbit secondary antibodies (1 : 5000, Cat. BA‐1000; Vector Labs). Peroxidase reactions were performed using ECL (Cat. 32106; Thermo Scientific, Waltham, MA, USA).

### 2.8 Soft agar assays

Soft agar colony formation assays were performed using 5000 cells in 0.4% agar on a base layer of 0.5% agar in DMEM. Cells were dosed immediately with doxycycline and were allowed to grow for 21 days. Colonies were stained using 0.005% crystal violet. The number of colonies was counted using imagej software (NIH, Bethesda, MD, USA) where clusters of cells 60 μm^2^ or greater were considered an individual colony. Three to six biological replicates were performed for each cell line. Protein was harvested from colonies as stated above, where the agar was first minced and softened via heat, and then, cells were filtered through a cell strainer to remove agar. Cells were lysed using standard RIPA buffer.

### 2.9 Orthotopic tumor assays

NSG (NOD.Cg‐*Prkdc* ^*scid*^ *Il2rg* ^*tm1Wjl*^/SzJ) or NRG (NOD.*Cg‐Rag1* ^*tm1Mom*^ *Il2rg* ^*tm1Wjl*^/SzJ) mice 8–20 weeks old were injected with 5.0 × 10^5^ cells in 20 μL PBS directly into the pancreas. Incisions were healed using sutures on the intraperitoneal cavity and staples to mend the skin wound. Mice were given acidified water and experimental mice were provided with gamma‐irradiated doxycycline chow replaced every 6 days (Cat. TD 08541, Envigo Laboratories, Madison, WI, USA). In some instances, mice were treated with gemcitabine (Cat. G6423; Sigma) where animals were IP injected with 25 mg·kg^−1^ every 3 days for 14 days. Each experimental group/time point consisted of 12–14 mice. All animal studies were conducted in strict compliance with the recommendations in the Guide for the Care and Use of Laboratory Animals of the National Institutes of Health and the Purdue University IACUC guidelines (Protocol Approval 1110000037).

### 2.10 Cell viability assays

Cell viability was assessed using the MTT assay (Cat. 11465007001, Roche). Panc‐1 Tet‐One, Tet‐MIST1, and Tet‐PTF1a cells were seeded at 5000 cells per well in 100 μL hgDMEM in a 96‐well plate. Cells were allowed to adhere for 4 h and then dosed with 2 μg·mL^−1^ doxycycline in 100 μL to bring the final concentration to 1 μg·mL^−1^ doxycycline. Twenty‐four hours postseeding, cells were dosed with increasing concentrations of gemcitabine from 1.0** **×** **10^−10^ to 1.0** **×** **10^−3^ m. Seventy‐two hours post‐gemcitabine treatment, 10 μL MTT was added to each well for a 4‐h period. Formazan was then solubilized using 100 μL solubilization reagent. Formazan product was measured at 560 nm in a 96‐well plate reader. GraphPad Prism was used to plot the data and calculate the log‐dose vs. response and the IC_50_ of each group. Four biological replicates were used for each data point.

### 2.11 Statistical analysis

Statistical significance was determined using unpaired one‐tailed *t*‐tests. In instances where samples displayed unequal variance (based on an *F*‐test), Welch's correction was applied. All *P*‐values ≤ 0.05 were considered statistically significant. Error bars represent the standard error of the mean. *P ≤ 0.05, **P ≤ 0.01, ***P ≤ 0.001.

### 2.12 Data accessibility

RNA‐Seq data have been deposited in the NCBI GEO database under accession number https://www.ncbi.nlm.nih.gov/geo/query/acc.cgi?acc=GSE106290.

## 3 Results

### 3.1 MIST1 and PTF1a loci are silenced early in the development of pancreatic cancer

**Figure 1:**
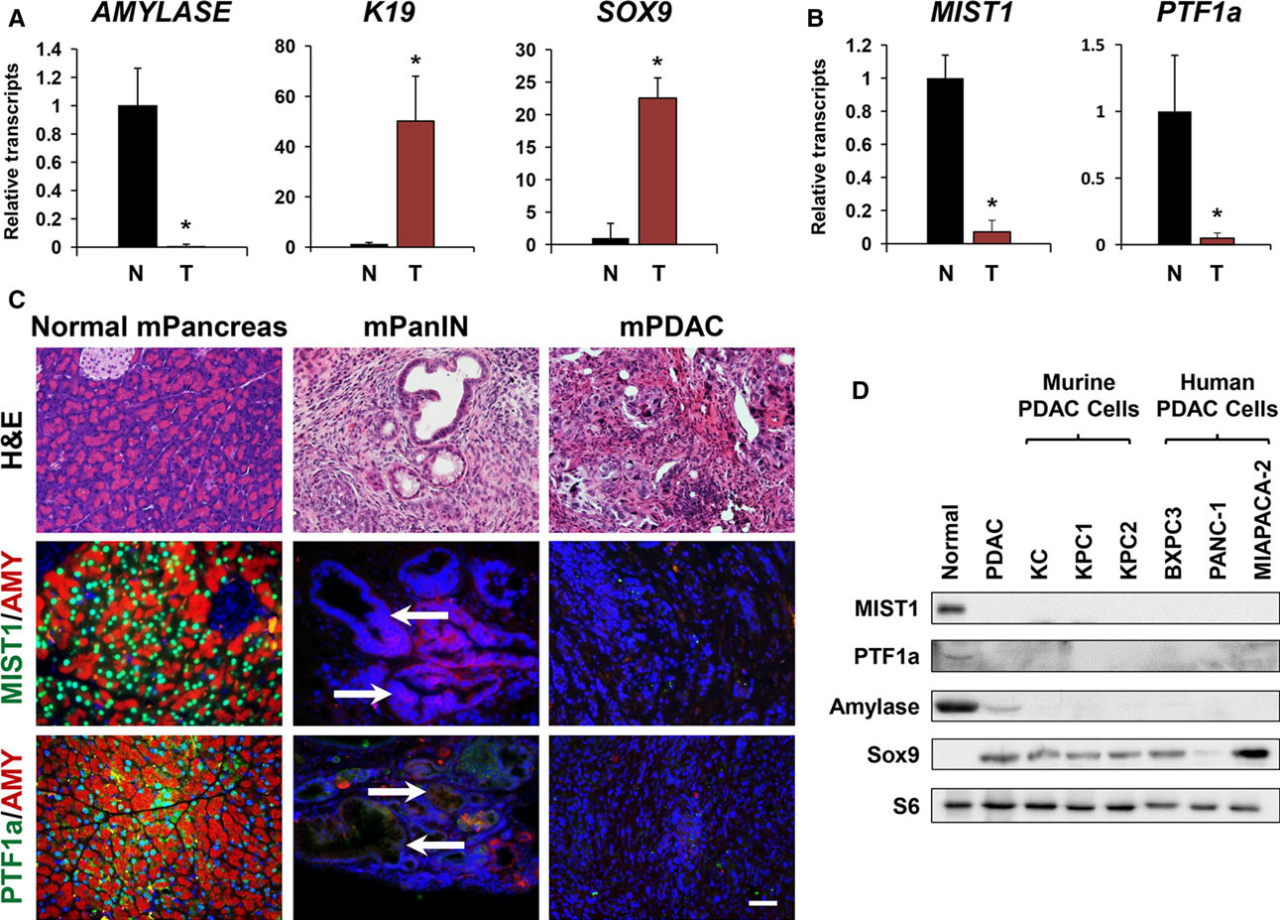
MIST1 and PTF1a expression in pancreatic cancer. (A) RT‐qPCR analysis of healthy adult murine pancreata (N) compared to murine KPC PDAC tumors (T). *Amylase*, a pancreatic acinar marker, is not expressed in PDAC tissue. Conversely, PDAC tissue has elevated transcript levels of the ductal genes *K19* and *SOX9* (*n* = 3 mice per data point). (B) *MIST1* and *PTF1a* transcripts are lost in KPC PDAC tumors (*n* = 3 mice per data point). (C) Tissue from KPC tumors stained with AMYLASE and MIST1 or AMYLASE and PTF1a. MIST1 and PTF1a are localized in the nuclei of normal acinar cells, but there is no protein detected in acinar‐derived PanIN (white arrows) and PDAC tissues (scale bar = 50 μm). Normal pancreas tissue is from 5‐month‐old control mice, PanIN tissue is from 11‐week‐old mice (21 days post‐tamoxifen), and PDAC tumors are from 5‐month‐old mice (4.1 months post‐tamoxifen). All histology is representative from 3 to 5 mice per disease state. (D) Immunoblot analysis of healthy adult murine pancreata compared to KPC PDAC tissue and a panel of mouse and human pancreatic cancer cell lines. MIST1 and PTF1a proteins are detected in normal pancreas tissue but lost in PDAC and all pancreatic cancer cell lines. *P ≤ 0.05.

A hallmark of PDAC is a dramatic shift in pancreas tissue architecture and gene expression. During tumorigenesis in mouse models of PDAC, quiescent acinar cells that synthesize and secrete large quantities of digestive hydrolases convert into tumor epithelial cells that acquire a duct‐like phenotype and activate genes normally associated with the ductal epithelium (Murtaugh and Leach, 2007; Storz, 2017; Wang *et al*., 2009). For example, in the *KPC* mouse model of PDAC (induced by acinar‐specific *Kras* ^*G12D*^ and *Trp53* ^*R172H*^ mutations), acinar cells transition into pancreatic intraepithelial neoplasia lesions (PanIN) that progress to PDAC (Habbe *et al*., 2008; Hruban *et al*., 2000; Murtaugh and Leach, 2007). During this transition, acinar‐specific genes (e.g., *AMYLASE*) become silenced, while ductal‐specific genes (e.g., *KERATIN19*) are rapidly induced (Fig. 1A) (Jonckheere *et al*., 2010; Karki *et al*., 2015). Driving these changes are key duct‐restricted transcription factors such as SOX9 (Fig. 1A) (Jacquemin *et al*., 2000; Kopp *et al*., 2012). Importantly, the silencing of two critical acinar‐restricted transcription factor genes – *MIST1* and *PTF1a* – is a common feature of PDAC tumors (Adell *et al*., 2000; Zhu *et al*., 2007) (Fig. 1B). Indeed, silencing *MIST1* and *PTF1a* loci is an early event as mouse ADM and PanIN lesions no longer accumulate MIST1 and PTF1a proteins when acinar cells transition toward a transformed phenotype (Fig. 1C). A similar loss of MIST1 and PTF1a proteins is observed in murine PDAC cell lines derived from *KC* and *KPC* mice (KC, KPC1, KPC2) as well as from human PDAC tumors (BxPC‐3, Panc‐1, MiaPaCa‐2) (Fig. 1D). Together, these results highlight a significant feature of acinar‐derived PDAC – silencing of the acinar transcription factor genes *MIST1* and *PTF1a*.

### 3.2 Generation of MIST1‐ and PTF1a‐induced Panc‐1 cell lines

Given the dramatic repression of *MIST1* and *PTF1a* expression in PDAC, we asked whether these key acinar bHLH factors could retain functional transcriptional activity in the context of a transformed PDAC cell. If transcriptional activity could be restored, we speculated that induced MIST1 or PTF1a might drive PDAC cells toward their original acinar‐like state. To test this hypothesis, we took advantage of a bidirectional inducible vector (Tet‐One) in which *MIST1* ^*myc*^ or *PTF1a* ^*my*c^ cDNA were inserted downstream of a tetracycline‐regulated promoter (Fig. S1A). Tet‐MIST1 and Tet‐PTF1a transgenes were introduced into human PDAC Panc‐1 cells, and transgene expression was activated by incubation of stable cell lines with doxycycline (Dox). As expected, MIST1 and PTF1a proteins were not detected in untreated cells (Fig. S1B,C). However, by 4 h post‐Dox addition, prominent MIST1 and PTF1a proteins were readily observed in the corresponding Tet‐MIST1 and Tet‐PTF1a lines, with maximal expression occurring by 24 h post‐Dox treatment (Fig. S1B,C). This level of expression remained stable in the presence of Dox for at least 21 days, the longest time examined (data not shown). Immunofluorescence analysis confirmed that greater than 85% of the Panc‐1 Tet‐MIST1 and Tet‐PTF1a cells expressed their respective proteins (Fig. S1B,C). Additionally, both factors exhibited transcriptional activity as each line activated specific target reporter genes (Fig. S1B,C).

### 3.3 MIST1 and PTF1a activate their respective acinar gene programs in PDAC cells

**Figure 2:**
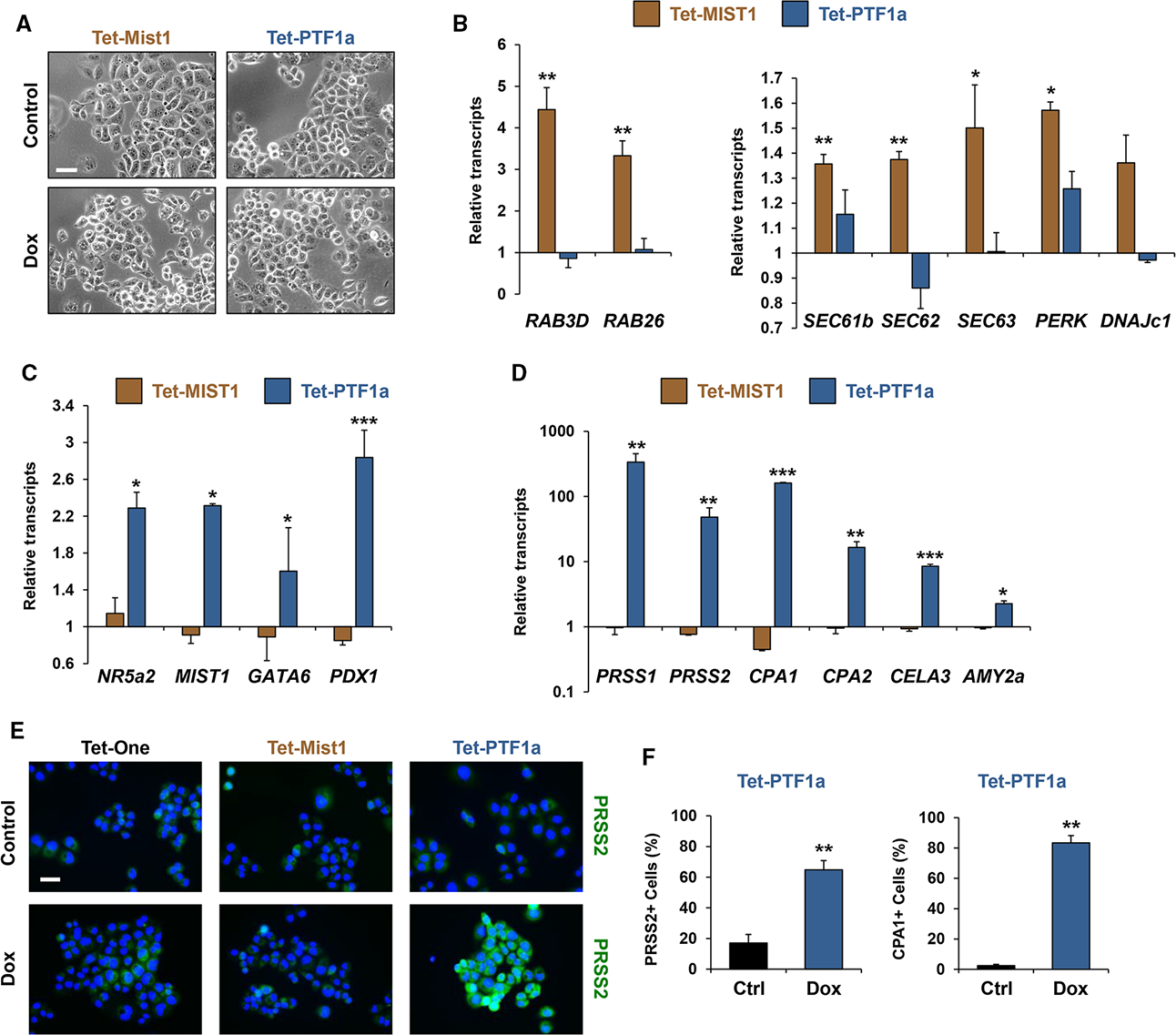
MIST1 and PTF1a activate known target genes in PDAC cells. (A) Phase contrast images of Panc‐1 Tet‐MIST1 and Tet‐PTF1a cells ± Dox (scale bar = 50 μm). (B) RT‐qPCR analysis of Panc‐1 Tet‐MIST1 and Tet‐PTF1a cells. Data are normalized to the same cell lines without doxycycline treatment and displayed as fold induction. MIST1 vesicle trafficking target genes *RAB3D* and *RAB26* and ER chaperones *SEC61b, SEC62, SEC63, PERK,* and *DNAJc1* are induced upon MIST1 expression. (C) Expression of acinar transcription factors *NR5a2, MIST1, GATA6, and PDX1* is induced in PDAC cells upon PTF1a induction. (D) Pancreas digestive enzymes *PRSS1, PRSS2, CPA1, CPA2, CELA3b,* and *AMY2a* transcripts accumulate upon PTF1a expression. (E) Trypsinogen (PRSS2) staining of Panc‐1 Tet‐One, Tet‐MIST1, and Tet‐PTF1a cells 72 h post‐doxycycline treatment (scale bar = 50 μm). (F) Percentage of Tet‐PTF1a cells expressing PRSS2 or carboxypeptidase (CPA1) upon doxycycline treatment. *P ≤ 0.05, **P ≤ 0.01, ***P ≤ 0.001.

To verify that the Panc‐1 Tet‐MIST1 and Tet‐PTF1a lines respond to activation of these key transcription factors, we examined the response of known MIST1 and PTF1a gene targets in the Panc‐1 cells treated with Dox, where the cells failed to exhibit any overt morphological changes upon Dox treatment (Fig. 2A). MIST1 is responsible for organizing acinar cell structure and regulated exocytosis, and part of this function is to activate expression of individual *RAB* genes to facilitate organelle and zymogen granule movement (Hou *et al*., 2015; Tian *et al*., 2010). As shown in Fig. 2B, MIST1 readily induced expression of *RAB3D* and *RAB26*, whereas induced PTF1a had no effect on these genes. Likewise, MIST1 promoted expression of the MIST1 target genes associated with the UPR pathway, including *SEC61b*,* SEC62*,* SEC63*,* PERK*, and *DNAJc1* (Fig. 2B) (Direnzo *et al*., 2012; Hess *et al*., 2011, 2016). Importantly, these data suggest that MIST1 can access target gene promoters in pancreatic cancer cells despite the inherent mutations, genomic instability, and altered epigenetic marks that are associated with these transformed cells (Deer *et al*., 2010; Fazio *et al*., 2017; Mehmood *et al*., 2014; Pin *et al*., 2015; Tan *et al*., 2009).

A similar response was observed in the Panc‐1 Tet‐PTF1a cells. PTF1a is a master regulator of acinar cell development and as such orchestrates expression of key acinar transcription factors including *NR5a2*,* RBPJL, GATA6*, and *PDX1* (Jiang *et al*., 2016; Krah *et al*., 2015; Thompson *et al*., 2012; Topno *et al*., 2017). As predicted, induced PTF1a in Panc‐1 cells produced significant increases in the downstream transcription factors *NR5a2*,* MIST1*,* GATA6*, and *PDX1* (Fig. 2C). In contrast, induced MIST1 expression failed to influence expression of this gene set. We next examined downstream hydrolase genes to determine whether they were similarly influenced by induced PTF1a expression. Although MIST1 failed to induce expression of this gene set, PTF1a readily activated expression of a large number of digestive enzyme genes including trypsinogens (*PRSS1*,* PRSS2*), carboxypeptidases (*CPA1*,* CPA2*), elastase (*CELA3*), and amylase (*AMY2a)* (Fig. 2D). Importantly, the response to induced PTF1a was also observed when cells were stained for PRSS2 and CPA1 protein, where up to 83% of the cells accumulated digestive enzymes (Fig. 2E,F). In contrast, control Tet‐One and Tet‐MIST1 cells failed to show evidence of PRSS2 and CPA1 protein output (Fig. 2E). In all cases, Tet‐MIST1 and Tet‐PTF1a and their respective target genes responded to Dox in a dose‐dependent fashion with maximal levels of products obtained with 1 μg·mL^−1^ Dox treatment (Fig. S1D,E).

Given the induced expression of MIST1 and PTF1a gene targets in human Panc‐1 cells, we next asked how Panc‐1 expression levels compared to gene transcript levels in human pancreas samples. Several RT‐qPCR analyses were performed, comparing Panc‐1 Tet‐MIST1 ± Dox, Panc‐1 Tet‐PTF1a ± Dox, and human pancreas RNA. As shown in Fig. S2A,B, most of the induced genes in Panc‐1 Tet‐MIST1 and Tet‐PTF1a cells were expressed to 20–40% levels of human pancreatic acinar cells, a remarkably robust response considering that the Panc‐1 line is a well‐established PDAC model. The only exception was that Tet‐PTF1a cells expressed significantly lower levels of digestive hydrolase genes (*PRSS1*,* CPA1*) when compared to intact pancreata. This is an interesting finding which suggests that although PTF1a can activate aspects of the PTF1a transcriptome in human PDAC cells, there remain limitations to the robustness of the responses. Indeed, it is possible that PTF1a primarily activates pancreas progenitor genes (e.g., *NR5a2*,* PDX1*) but not classic differentiation genes (e.g., *PRSS1*) to the maximal levels found in patient pancreata.

We next examined whether the ability of PDAC cells to respond to Tet‐MIST1 and Tet‐PTF1a was unique and/or restricted to only Panc‐1 cells. For these studies, we generated new Tet‐MIST1 and Tet‐PTF1a lines using human BxPC‐3 PDAC cells and mouse KPC PDAC cells that were derived by activating mutations in adult acinar cells. As predicted, BxPC‐3 and KPC cells activated expression of the corresponding MIST1 and PTF1a target genes only in the presence of Dox (Fig. S3A,B) in an analogous fashion as shown for Panc‐1 cells. Thus, expression of MIST1 and PTF1a leads to universal cell responses. Together, these data reveal that PTF1a and MIST1 retain functional capacity in pancreatic cancer cells and can activate their respective transcriptional networks, providing evidence that genes involved in acinar development can be reactivated in human and mouse PDAC cells.

### 3.4 MIST1 and PTF1a promote similar but distinct acinar‐associated pathways in PDAC cells

**Figure 3:**
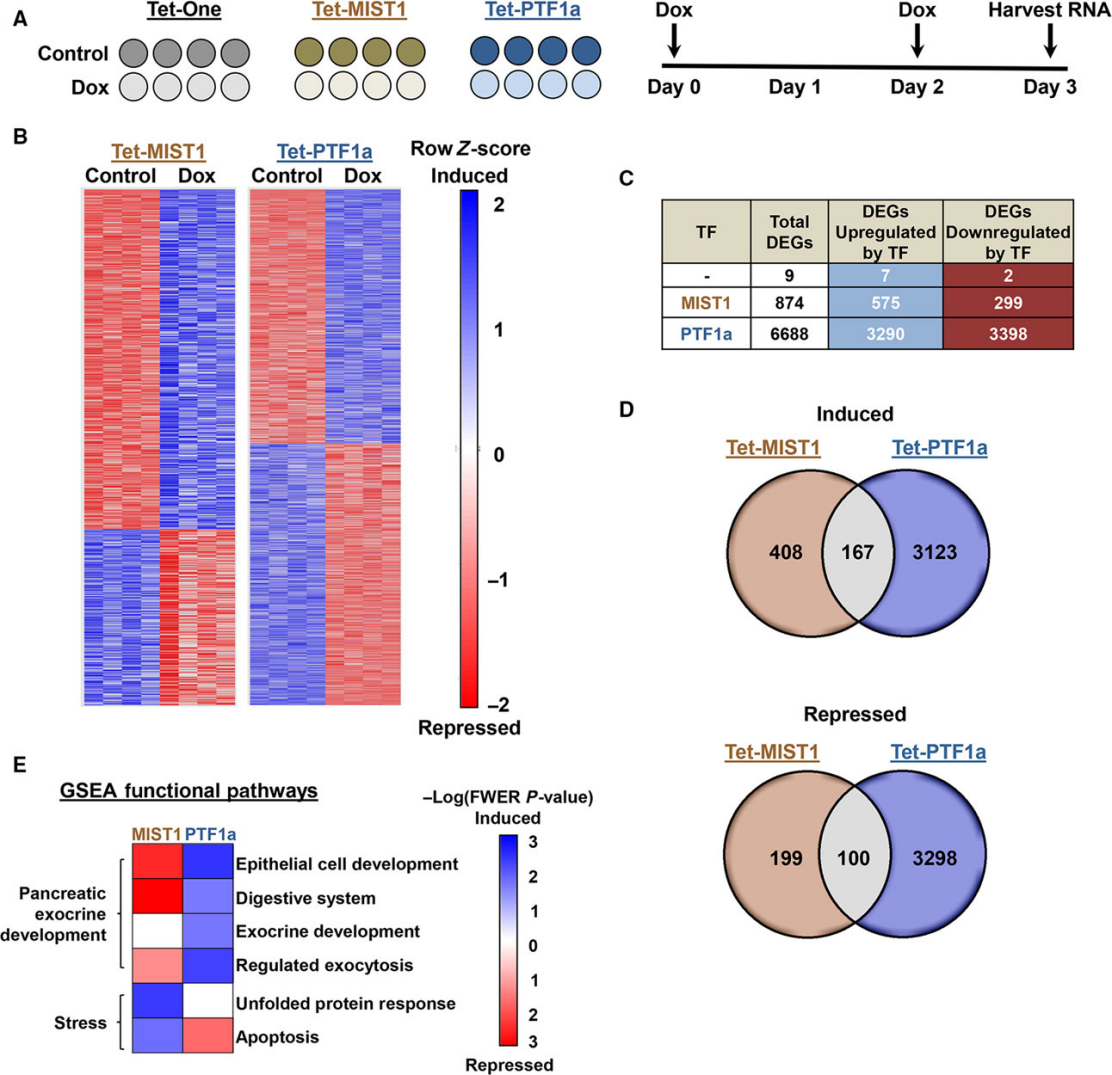
RNA sequencing of Tet‐MIST1 and Tet‐PTF1a. (A) Experimental strategy and timeline for cells subjected to RNA‐Seq. (B) Representative heatmaps of four control samples compared to four doxycycline‐treated samples for the Tet‐MIST1 and Tet‐PTF1a cells. Heatmaps depict the comparison of log2‐transformed read counts for each gene. (C) A total of 874 genes are differentially expressed in the Tet‐MIST1 cell line, while 6688 genes were differentially expressed in Tet‐PTF1a cells. (D) Venn diagrams comparing induced and repressed genes in Tet‐MIST1 vs. Tet‐PTF1a samples. (E) Summary heatmap displaying −Log (FWER *P*‐value) for biological and molecular pathways identified by GSEA.

MIST1 and PTF1a retain functional activity in the context of PDAC cells as they each promote expression of their known pancreatic acinar gene networks. To obtain a broader profile of the effects of induced expression of MIST1 and PTF1a in PDAC, RNA‐Seq studies were conducted on Panc‐1 Tet‐One, Tet‐MIST1, and Tet‐PTF1a cells ±Dox treatment (Fig. 3A). As predicted, control Tet‐One cells exhibited minor responses, displaying only nine DEGs when transcript profiles were compared in ±Dox‐treated groups (Fig. 3C). In contrast, 874 DEGs were identified in the Tet‐MIST1 line (Fig. 3B,C). A similar but more robust gene expression response was observed with Tet‐PTF1a cells where 6688 DEGs were identified (Fig. 3B,C). The sevenfold difference in DEGs between MIST1 and PTF1a cells is likely due to the functional nature of each transcription factor, where PTF1a is a master regulator for pancreatic acinar development while MIST1 is considered a scaling factor, augmenting specific transcription programs (Hess *et al*., 2016; Jiang *et al*., 2016; Kondratyeva *et al*., 2017; Mills and Taghert, 2012; Tian *et al*., 2010). Nonetheless, there was considerable overlap in DEGs regulated by both transcription factors, with 167 target genes induced by PTF1a and MIST1, representing 29% of the upregulated MIST1 targets (Fig. 3D). Likewise, 33% of MIST1 repressed targets were shared with PTF1a (Fig. 3D).

As transcriptional activators, PTF1a and MIST1 likely act directly on a subset of the induced DEGs which in turn produce additional downstream changes in gene expression. Interestingly, PTF1a and MIST1 motifs were more significantly enriched in the promoter regions of induced DEGs compared to repressed genes (Fig. S4A) although the biological relevance of these sites, as well as motifs discovered *de novo* (Doc. S1), will require further analysis. Nonetheless, to gain additional insight into possible direct PTF1a‐ and MIST1‐regulated genes within our RNA‐Seq data, DEGs were compared to previously published PTF1a ChIP‐Seq (https://www.ncbi.nlm.nih.gov/geo/query/acc.cgi?acc=GSE86262) and MIST1 ChIP‐Seq (https://www.ncbi.nlm.nih.gov/geo/query/acc.cgi?acc=GSE86289) mouse studies (Hoang *et al*., 2016; Jiang *et al*., 2016a). As predicted, a similar proportion of induced and repressed DEGs were found to have ChIP‐Seq peaks in near proximity with several well‐characterized PTF1a‐ and MIST1‐regulated genes highlighted in the overlap between the ChIP‐Seq and induced DEGs (Fig. S4B) (Ahnfelt‐Rønne *et al*., 2012; Direnzo *et al*., 2012; Hoang *et al*., 2016; Jiang *et al*., 2016a; Lo *et al*., 2017; Wiebe *et al*., 2007). This discovery further shows that PTF1a and MIST1 maintain regulation of their transcriptional networks, even in the context of PDAC. Indeed, KEGG pathway analyses showed enrichment for ER protein processing and protein export in the PTF1a‐ and MIST1‐induced DEGs, respectively, including several known direct target genes in both categories (Fig. S5A–D).

Gene Set Enrichment Analysis, which is capable of analyzing each dataset as a whole, was also performed taking into account the directionality of DEG expression. As expected, a wide range of cellular processes involved in pancreatic development and function were identified. For example, MIST1 promoted expression of genes involved in stress responses, including key genes of the UPR and apoptosis pathways (Fig. 3E), which agrees with previous studies showing that MIST1 functions to alleviate UPR stress by increasing expression of chaperones and other UPR genes (Hess *et al*., 2011, 2016; Jiang *et al*., 2016). Surprisingly, MIST1 expression in Panc‐1 cells negatively influenced genes involved in exocrine development (Fig. 3E), suggesting that MIST1 may function differently in PDAC cells compared to embryonic and adult acinar cells (Direnzo *et al*., 2012; Jiang *et al*., 2016; Karki *et al*., 2015; Pin *et al*., 2001). In contrast, PTF1a exhibited a more potent transcriptional response with a number of enriched pathways being induced that are involved in pancreatic exocrine development, including ‘Digestive System’, ‘Epithelial Cell Development’, and ‘Exocrine Development’ (Fig. 3E).

### 3.5 PDAC‐associated pathways are negatively influenced by MIST1 and PTF1a expression

**Figure 4:**
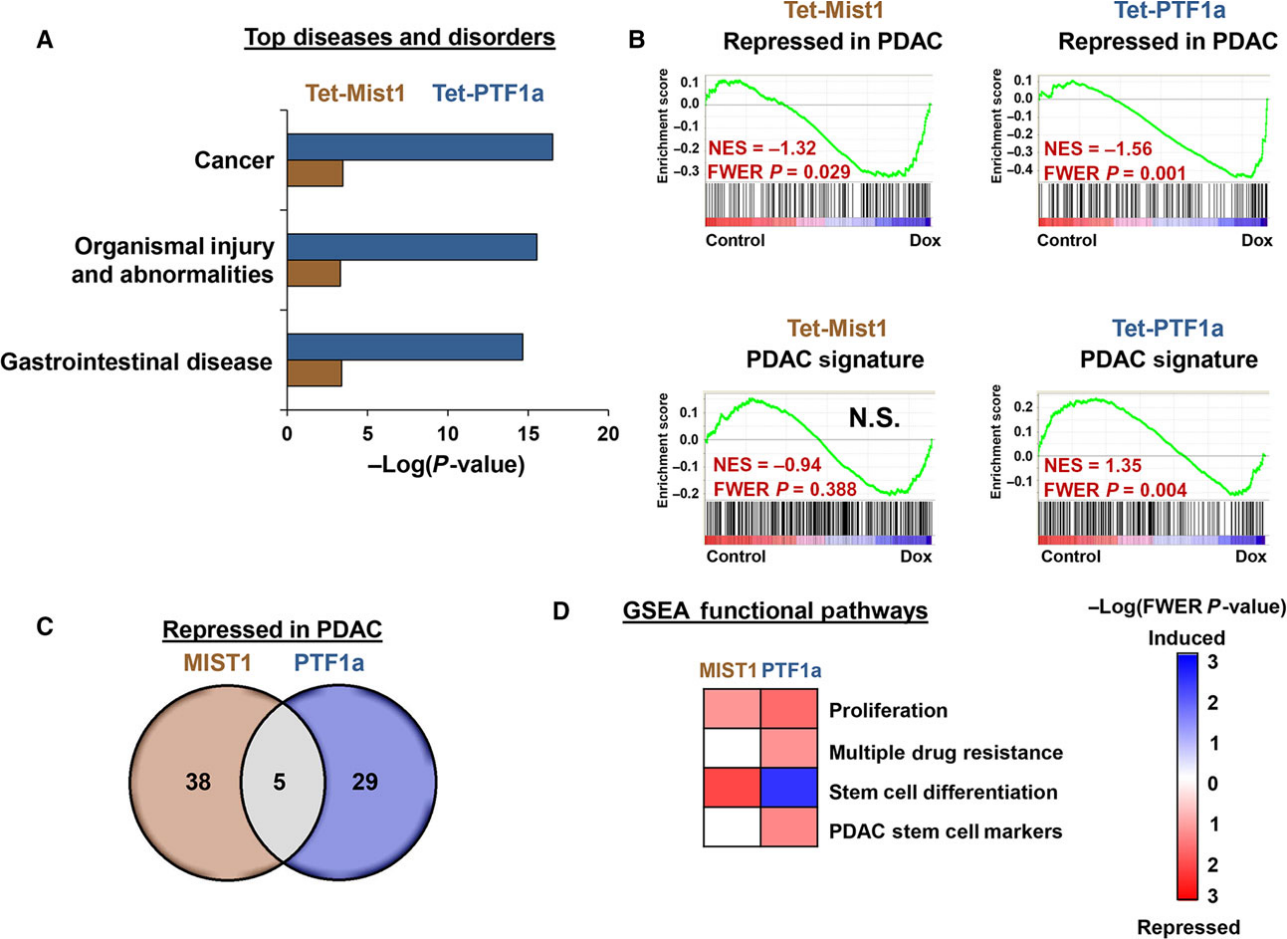
Tet‐MIST1 and Tet‐PTF1a gene expression analysis. (A) Top three disease pathways identified by IPA. (B) GSEA of genes associated with pancreatic cancer using the ‘Gruetzman Pancreatic Cancer Up’ list (PDAC Signature) and genes classically repressed in PDAC (‘Gruetzman Pancreatic Cancer Down’ list). (C) Venn diagram comparison of genes differentially regulated in ‘repressed in PDAC’. (D) Heatmaps of disease‐associated pathways from GSEA altered upon MIST1 or PTF1a induction. N.S., not significant.

Ingenuity pathway analysis identified ‘cancer’ as the top disease pathway altered in both Tet‐MIST1 and Tet‐PTF1a cell lines, followed by ‘organismal injury and abnormalities’ and ‘gastrointestinal disease’ (Fig. 4A, Doc. s2). In all three cases, PTF1a exhibited a greater footprint within the pathways than MIST1, again demonstrating the wider transcriptional activity and gene expression responses of this bHLH factor. Identification of ‘cancer’ pathways suggested that tumor‐associated properties may be decreased when MIST1 and PTF1a are induced in PDAC cells. GSEA revealed that genes which are classically repressed in PDAC were significantly induced upon bHLH induction (Fig. 4B). Similarly, PTF1a expression negatively influenced genes associated with a ‘PDAC signature’ (Fig. 4B). Interestingly, there was little overlap in DEGs in the ‘repressed in PDAC’ signature when comparing MIST1 and PTF1a, suggesting that different MIST1 and PTF1a target genes are involved in affecting PDAC properties (Fig. 4C). Consistent with this assumption, we observed several antitumorigenic trends in PTF1a cells which were not seen with MIST1, including increased ‘stem cell differentiation’ and decreased ‘PDAC stem cell markers’ and ‘multiple drug resistance’ pathways (Fig. 4D).

### 3.6 PTF1a induction reduces cancer stem cell characteristics

**Figure 5:**
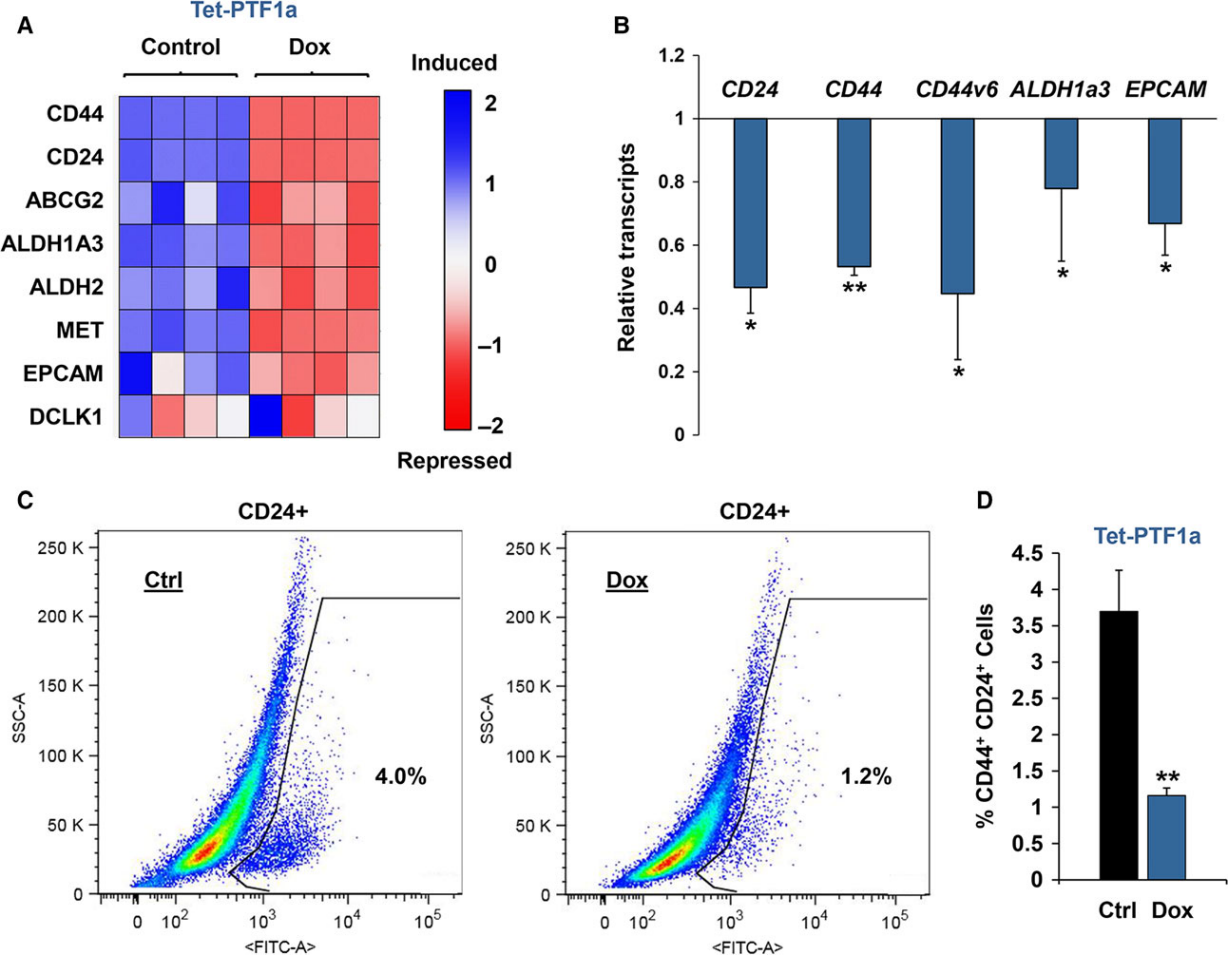
PTF1a expression decreases CSC properties. (A) Heatmap of a curated list of pancreatic CSC markers in Tet‐PTF1a cells ± Dox. (B) RT‐qPCR of selected pancreatic CSC markers. (C) Flow cytometry of CD24+ Tet‐PTF1a cells ± Dox. (D) CD44+/CD24+ cells quantified from Tet‐PTF1a cells ± Dox. *P ≤ 0.05, **P ≤ 0.01.

**Figure 6:**
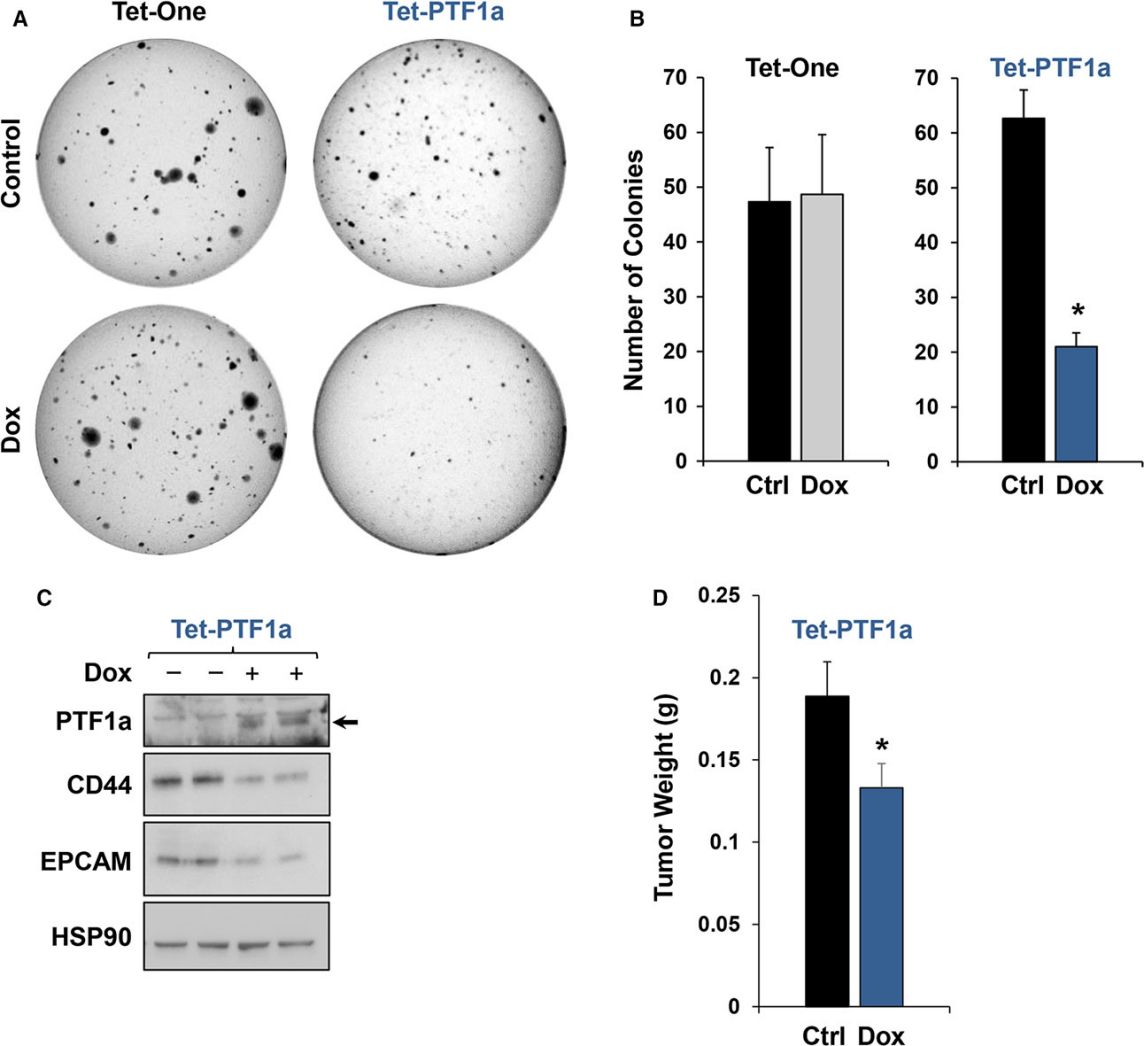
PTF1a expression decreases soft agar colony and orthotopic pancreatic cancer tumor growth. (A,B) Soft agar colony formation assay of Tet‐One and Tet‐PTF1a cells ± Dox. (C) Immunoblot analysis of CD44 and EPCAM from soft agar colonies of Tet‐PTF1a cells ± Dox. CD44 and EPCAM show marked reductions in the PTF1a expressing colonies. (D) Orthotopic xenograft assays testing Panc‐1 Tet‐PTF1a tumor growth in mice subjected to ± Dox chow. *P ≤ 0.05.

GSEA suggested that induced PTF1a activity promotes decreased CSC properties. To examine this further, we curated a list of eight (*CD24, CD44, EPCAM, ABCg2, ALDH1a3, MET, DCLK1, ALDH2*) pancreatic stem cell markers and examined their overall expression in our RNA‐Seq dataset. Interestingly, PTF1a induction significantly reduced seven of the eight CSC markers, with the only exception being *DCLK1* (Fig. 5A). Consistent with these findings, RT‐qPCR analysis confirmed that *CD24*,* CD44, CD44v6, ALDH1a3,* and *EPCAM* transcripts were all significantly decreased upon PTF1a induction (Fig. 5B). In contrast, MIST1 induction had little to no effect on CSC marker expression (data not shown). Flow cytometry corroborated our initial findings, revealing ~ 70% reduction in CD24+/CD44+ cells upon PTF1a expression (Fig. 5C,D). The decrease in CD24+/CD44+ cells also translated into a substantial reduction in soft agar colony formation when Tet‐PTF1a cells were induced to express PTF1a (Fig. 6A,B). As predicted, the reduced colony formation was reflected in decreased CD44 and EPCAM protein levels (Fig. 6C). Importantly, Dox treatment had no effect on colony formation in the Panc‐1 Tet‐One cells (Fig. 6A,B) or with parental Panc‐1 cells (data not shown). Finally, to assess the ability of CSC properties to support tumor initiation and growth *in vivo*, we performed pancreatic orthotopic tumor assays. As shown in Fig. 6D, a 30% reduction in tumor growth was consistently observed when mice were maintained on a Dox diet. Collectively, these data provide evidence that induced expression of PTF1a reduces pancreatic CSC properties and overall PDAC tumor growth in orthotopic assays.

### 3.7 PTF1a sensitizes PDAC cells to gemcitabine treatment

**Figure 7:**
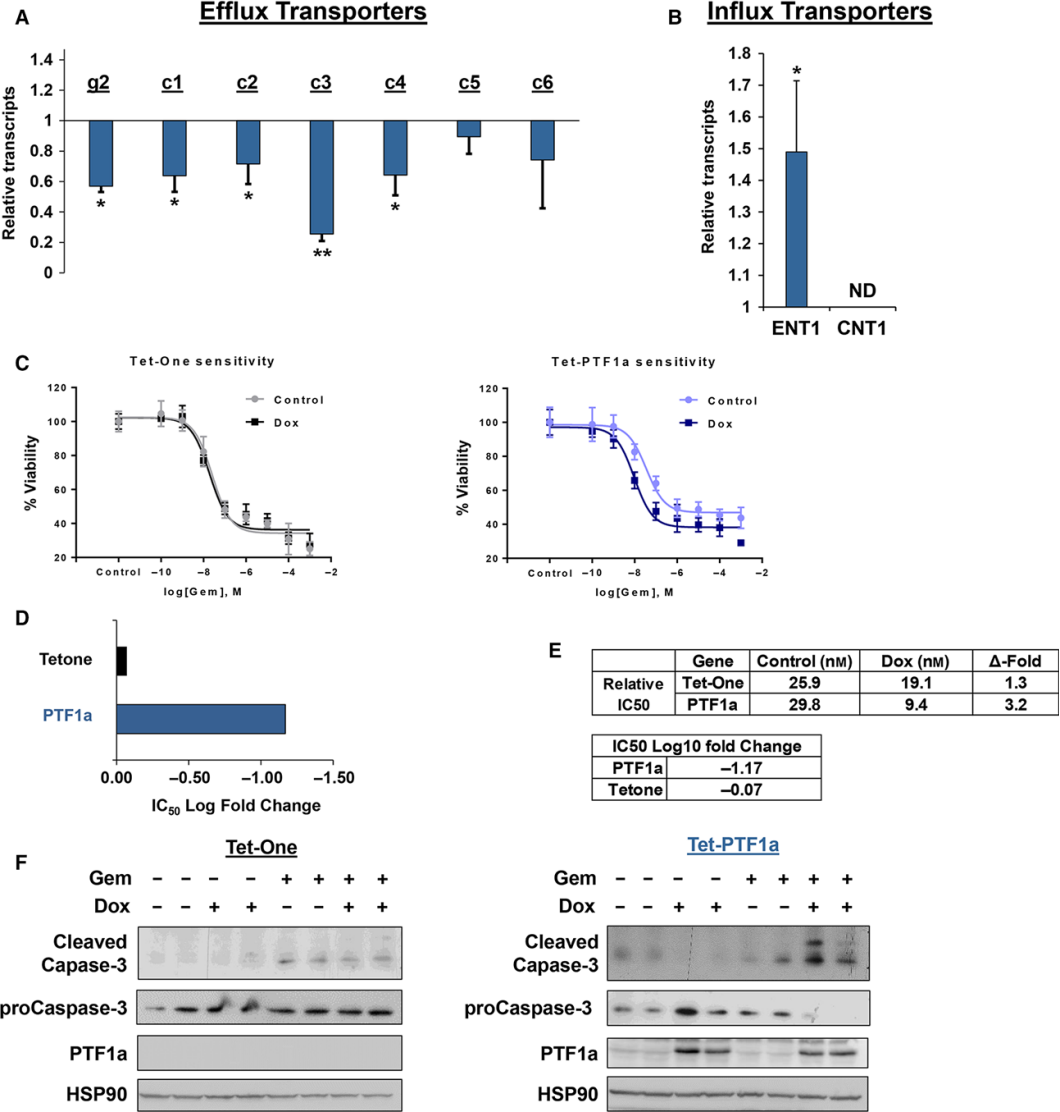
PTF1a sensitizes Panc‐1 cells to gemcitabine treatment. (A) RT‐qPCR of gemcitabine efflux transporters for Tet‐PTF1a cells ± Dox. (B) RT‐qPCR of gemcitabine influx transporters *ENT1* and *CNT1*. (C) IC_50_ kill curve of Tet‐inducible cell lines using a gemcitabine gradient for 72 h. (D,E) Log_10_ fold change of IC_50_ when cells are treated with doxycycline to induce PTF1a. (F) Immunoblot analysis of Tet‐inducible cells using 10 μm gemcitabine for 72 h. Cleaved caspase‐3 is elevated upon PTF1a induction, while Tet‐One cells display no change. *P ≤ 0.05, **P ≤ 0.01. ND, not detected.

A key feature of CSCs is the ability to sustain multidrug resistance. Given that PTF1a expression leads to a decreased stem cell population, we asked whether PTF1a activity also altered CSC characteristics. As a first step, we screened expression profiles of ATP‐binding cassette (ABC) efflux transporters known to be critical to clinical PDAC drug resistance because they pump gemcitabine out of cells (Dean, 2009; Ding *et al*., 2010; Moitra, 2015; Tanaka *et al*., 2011). Notably, five transporter genes showed decreased transcript levels following PTF1a activity, while expression of the remaining three genes was either undetected (*ABCB1*) or showed a similar (but statistically insignificant) decrease in expression upon PTF1a induction (Fig. 7A). As predicted, Tet‐MIST1 and Tet‐One control cells did not alter expression of any of the ABC efflux transporters (data not shown). Interestingly, the gemcitabine importer *ENT1* also exhibited a significant change in gene expression upon PTF1a induction, but in this case expression was elevated (Fig. 7B). Taken together, these results suggest that the steady‐state level of gemcitabine in the cytosol may be altered in the presence of PTF1a. Indeed, direct testing of gemcitabine on the Panc‐1 Tet‐PTF1a cells revealed an increased cell death efficacy in cells expressing PTF1a when compared to control cells (Fig. 7C), with an order of magnitude increase in sensitivity to gemcitabine upon PTF1a induction (Fig. 7D) and a threefold lower IC_50_ value for these cells when treated with Dox (Fig. 7E). As predicted, cleaved caspase‐3 was also elevated in the PTF1a‐expressing cells when treated with gemcitabine compared to control cells treated with gemcitabine alone (Fig. 7F).

**Figure 8:**
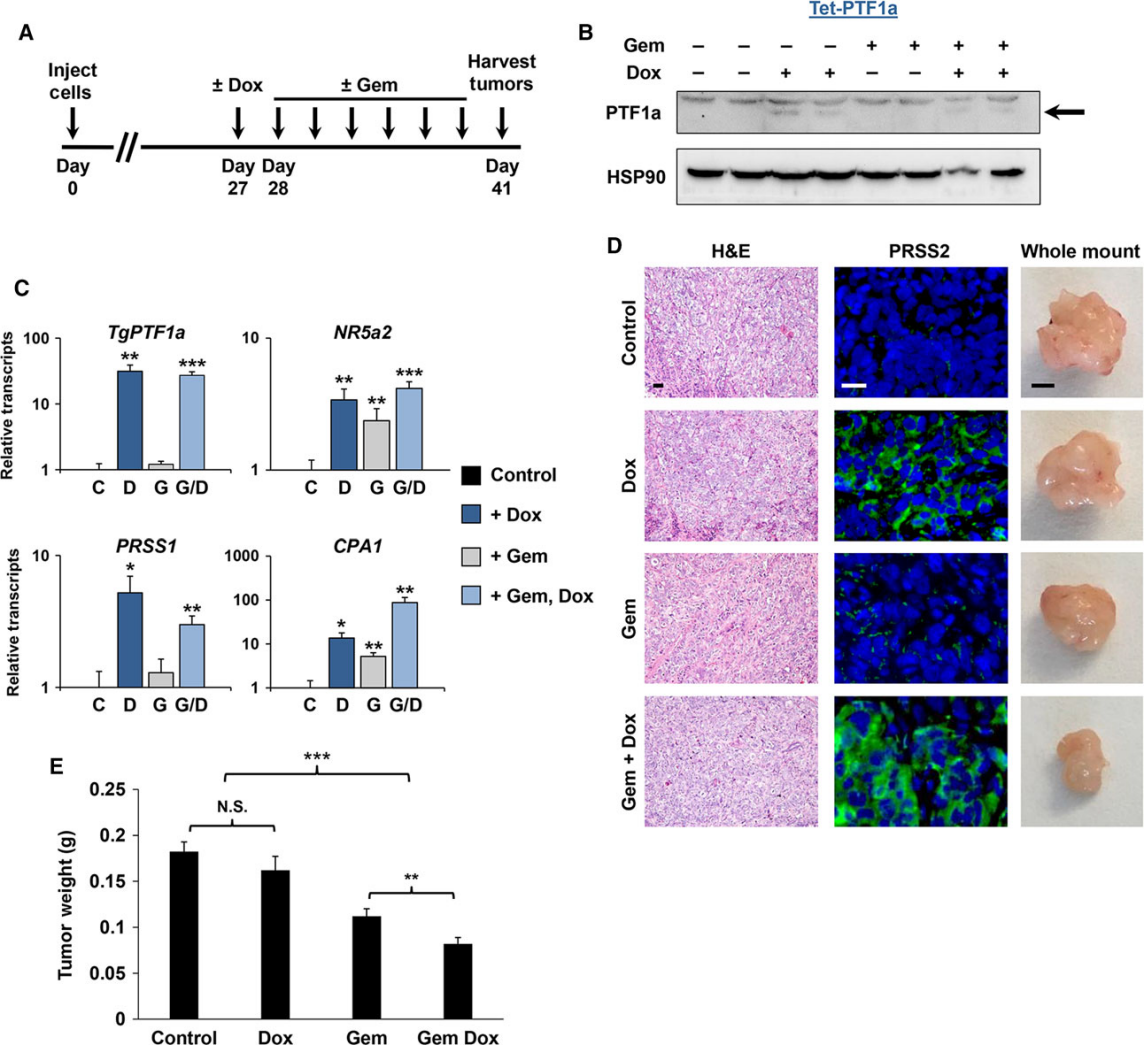
PTF1a sensitizes PDAC cells to gemcitabine *in vivo*. (A) Experimental timeline. (B) Immunoblot of PTF1a expression from excised tumors in mice treated ± Dox and ± Gem. (C) RT‐qPCR of tumor samples from the indicated groups for the Tet‐induced PTF1a transgene (*TgPTF1a*) as well as for endogenous *NR5a2*,*PRSS1*, and *CPA1* genes. (D) Hematoxylin and eosin (H&E) staining, PRSS2 immunofluorescence (green), and whole‐mount images of tumors isolated from mice treated ± Dox and ± Gem (scale bars = 50 μm for sections; 2.5 mm for whole‐mount images). (E) Tumor weights obtained from Panc‐1 Tet‐PTF1a cells ± Dox and ± Gem (*n *= 12–14 mice per experimental group). *P ≤ 0.05, **P ≤ 0.01, ***P ≤ 0.001, N.S., not significant.

As PTF1a expression resulted in increased sensitivity to gemcitabine, we next tested the ability of induced PTF1a to increase tumor sensitivity to gemcitabine *in vivo*. Panc‐1 Tet‐PTF1a cells were orthotopically injected into immunocompromised mice and allowed to grow for 27 days, at which time the mice were randomly assigned to groups receiving either no treatment, Dox only, gemcitabine only, or Dox and gemcitabine together. Gemcitabine was administered every 3 days for the following 14‐day time span (Fig. 8A). This strategy permitted an assessment of PTF1a's ability to sensitize an already established PDAC tumor. As expected, PTF1a expression was observed solely in the +Dox groups (Fig. 8B,C). Similarly, PTF1a target genes (*NR5a2*,* PRSS1*,* CPA1*) exhibited the predicted induction in the +Dox samples (Fig. 8C). Despite H&E sections failing to reveal any distinct morphological differences between groups, PRSS2 protein accumulation was readily detected and restricted to samples expressing PTF1a (Fig. 8D). Analysis of control vs. +Dox groups revealed that inducing PTF1a expression in established tumors produced only minor and statistically insignificant decreases in tumor burden (Fig. 8D,E). Treatment with gemcitabine alone resulted in a 38% decrease in tumor burden in cells not expressing PTF1a. However, when established tumors were provided both Dox (to activate PTF1a) and gemcitabine, total tumor burden decreased by 55%, a 1.4‐fold enhancement in gemcitabine sensitivity (Fig. 8D,E). These results support the concept that induced PTF1a sensitizes PDAC cells to gemcitabine treatment.

## 4 Discussion

Pancreatic cancer is a lethal disease that has failed to be therapeutically controlled, despite enormous research and clinical efforts. Recent studies utilizing mouse models have revealed that adult duct cells expressing *Hnf1b* or *Sox9* can serve as the cell of origin to PDAC if the cells are subjected to activating *Kras* mutations and inactivating mutations in TP53 (deletion or *Tp53* ^*R172H*^) (Bailey *et al*., 2016a; Lee *et al*., 2018). Another PDAC progression model suggests that the disease can originate from acinar cells harboring an oncogenic *Kras* mutation (Collins *et al*., 2012, 2014; Habbe *et al*., 2008; Kopp *et al*., 2012; Krah *et al*., 2015; Shi *et al*., 2009, 2013). Interestingly, despite both duct and acinar cells involved in disease initiation, the cell of origin can differentially affect tumor development schemes (Bailey *et al*., 2016a; Lee *et al*., 2018). Nonetheless, the well‐described acinar → PDAC progression model provides opportunities to develop new strategies to interrupt key processes along the entire disease spectrum and reverse the malignant properties of PDAC cells. Indeed, work from the Pasca di Magliano Lab (Collins *et al*., 2012, 2014) has shown that precancerous PDAC lesions exhibit plasticity and can be reverted back to healthy pancreatic acinar tissue upon loss of oncogenic KRAS signaling. These studies have provided a key insight into possibilities for reprogramming PDAC cells.

A complementary approach to reversing KRAS signaling in PDAC cells is to manipulate transcriptional networks to drive cells toward a nontumorigenic phenotype. Acinar cells have been particularly amenable to reprogramming as these cells convert to duct‐like cells during ADM and PDAC development through a SOX9‐dependent network (Kopp *et al*., 2012). This phenomenon is driven in part by upregulation of ERBB2 expression and elevated ERBB signaling (Grimont *et al*., 2014). Additionally, acinar cells convert to insulin‐expressing β‐cells when the transcription factors NGN3, PDX1, and MAFA are exogenously expressed (Zhou *et al*., 2008). A similar reprogramming phenomenon has been observed in PDAC cells engineered to express elevated levels of the bHLH factor E47 (Kim *et al*., 2015). Increased E47 shifts the bHLH:ID axis in these cells, ultimately activating acinar cell properties and decreasing tumorigenic characteristics, providing one of the first reports that PDAC cells are susceptible to alterations in their transcription networks. In this study, we tested whether other bHLH transcription factors could be instrumental in driving PDAC cells toward their acinar origin and subsequently decrease cancer‐associated properties. We chose to focus on two key pancreas bHLH factors (MIST1 and PTF1a) because (i) MIST1 and PTF1a are silenced in KRAS‐expressing acinar cells, (ii) their silencing promotes the progression of precancerous lesions (Krah *et al*., 2015; Shi *et al*., 2009), and (iii) forced expression of MIST1 in the context of KRAS signaling mitigates the effects of silencing the endogenous alleles on PDAC formation (Shi *et al*., 2013), suggesting that these bHLH factors function as tumor suppressors. Notably, both PTF1a and MIST1 are essential for the acinar gene network where PTF1a drives an acinar transcription system and expression of digestive enzyme genes while MIST1 regulates genes essential to acinar cell–cell communication, calcium flux, and acinar cell secretion (Beres *et al*., 2006; Direnzo *et al*., 2012; Garside *et al*., 2010; Hess *et al*., 2016; Jiang *et al*., 2016; Krah *et al*., 2015; Pin *et al*., 2001)

An important outcome from our study was that KRAS^G12D^, p53^R273H^, CDKN2A/p16^KO^ PDAC cells (Panc‐1) retain the capacity to activate the acinar gene program following forced expression of PTF1a or MIST1. Indeed, PDAC cells induced to express PTF1a or MIST1 promoted acinar‐specific gene expression. For example, induced expression of PTF1a activated a previously silent acinar transcription network that included triggering expression of the key transcription factor genes *NR5a2, GATA6, PDX1,* and *MIST1*, which subsequently led to elevated expression of acinar‐specific digestive hydrolase genes *PRSS1, PRSS2, CPA1, CPA2, CELA3b,* and *AMY2a*. Interestingly, although PTF1a activity in human PDAC cells induced expression of acinar transcription factors to 20–40% the levels found in normal, intact human pancreata, PTF1a was inefficient in inducing comparable levels of digestive enzyme genes (e.g., *PRSS1*). Many of the PTF1a‐induced transcription factors (NR5a2, GATA6, PDX1) are known to mark and be critical to pancreatic progenitor cells (Zhou *et al*., 2008). Thus, it remains possible that PTF1a primarily activates an early developmental program in PDAC cells which may help the cells achieve a more mature acinar phenotype. Similar results were obtained with induced MIST1 where a large number of MIST1 target genes, including *RAB3D, RAB26, SEC61b, SEC62,* and *SEC63*, were expressed in PDAC cells. Consistent with inducing a differentiated phenotype, MIST1 and PTF1a also activated genes that are normally repressed in cancer, including *NR5a2*,* SGK1*,* XBP1*,* SEC61b*,* DMPK*,* RBP1*, and *CLDN10* (Grützmann *et al*., 2005). However, in all cases PTF1a was more potent in decreasing cancer‐associated gene expression when compared to MIST1‐induced profiles, suggesting that PTF1a has a greater global impact in driving cells toward a quiescent, acinar‐specific phenotype. These findings are consistent with a number of studies showing that PTF1a functions as a transcriptional master regulator while MIST1 serves as a scaling factor, primarily augmenting gene expression patterns (Capoccia *et al*., 2013; Hess *et al*., 2016; Jiang *et al*., 2016; Krapp *et al*., 1996, 1998; Mills and Taghert, 2012). Thus, progenitor cell and acinar genes within PDAC cells remained accessible to activation by PTF1a and MIST1, revealing that PDAC cells are not irreversibly locked into an undifferentiated cancer cell state.

The phenomenon of MIST1 and PTF1a to reprogram the PDAC transcriptome was not restricted to Panc‐1 cells as similar results were obtained when human BxPC‐3 PDAC cells and mouse KPC PDAC cells were engineered to express induced MIST1 and PTF1a. This was an intriguing finding given that each cell line harbors unique oncogene and tumor suppressor gene mutations. Additionally, although Panc‐1 and BxPC‐3 cells are thought to be derived from duct cells, the KPC line was derived from acinar cells. Our results are particularly interesting given the recent discovery that human PDAC tumors can be classified into four different molecular subtypes (squamous; pancreatic progenitor; immunogenic; and aberrantly differentiated endocrine exocrine tumors) (Bailey *et al*., 2016b). Thus, despite potential differences in the molecular evolution and signature of PDAC subtypes, strategies to alter transcription networks may still be effective in most PDAC examples.

One of the more striking phenotypes associated with PTF1a expression in PDAC cells was the consistent decrease in CSCs and CSC properties. It is well established that pancreatic CSCs initiate and propagate PDAC tumor formation and are a major contributor to drug and radiotherapy resistance (Li *et al*., 2007; Vaz *et al*., 2014; Westphalen *et al*., 2016). Interestingly, PTF1a expression produced a marked reduction in expression of a number of CSC genes including transcript decreases in *CD24*,* CD44*,* CD44v6*,* ALDH1a3*, and *EPCAM*. Indeed, PTF1a induction generated a threefold reduction in CD44+/CD24+ CSCs as well as a threefold reduction in soft agar colony formation. The decrease in CSC numbers was also associated with 30% reductions in orthotopic tumor burden when mice injected with Tet‐PTF1a Panc‐1 cells were provided Dox. Thus, activating/inducing PTF1a in PDAC tumor cells may provide a new therapeutic venue to decrease PDAC progression by lowering CSC properties.

Given that CSCs are resistant to therapeutics, we also examined whether PTF1a expression sensitized cells to gemcitabine treatment, a standard therapy for patients with pancreatic cancer (Heinemann *et al*., 2000; Kumar Jena *et al*., 2012). PTF1a expression produced marked reductions in expression of a large family of ABC efflux transporters, suggesting that gemcitabine cannot be actively pumped out of PDAC cells that express PTF1a. Indeed, gemcitabine flux was significantly altered upon activation of PTF1a, resulting in a 17‐fold enhancement in gemcitabine efficacy when tested *in vitro*. A similar increase in efficacy was observed in pancreatic orthotopic tumor assays where PDAC cells expressing PTF1a exhibited significantly greater sensitivity to gemcitabine compared to controls. To our knowledge, these are the first studies to show that rescuing PTF1a expression in the context in PDAC cells provides a reduction in CSCs and increased sensitivity to gemcitabine treatment.

The ability to reprogram cells with key transcription factors has evolved rapidly since the pioneering studies of Takahashi and Yamanaka in generating iPS cells from adult somatic cells (Takahashi and Yamanaka, 2006). And although strategies to convert iPS cells into many different differentiated cell types are now commonplace (Shi *et al*., 2017), there has been limited progress in shifting transcription networks to restore normal cell properties to cancer cells. Many of the remaining obstacles concern reversing multiple mutations that exist in a heterogeneous tumor cell population (Bailey *et al*., 2016b). Nonetheless, we now show that (i) loci normally silent in PDAC cells can be reactivated when key transcription factors are present, (ii) activation of acinar gene expression profiles can have a profound effect on CSC properties, and (iii) induction of PTF1a leads to decreases in ABC efflux transporters, ultimately producing higher efficacy potential of antitumor therapeutics. Future challenges will be to develop strategies to activate expression of master regulator genes directly in tumor cells. Because *PTF1a* and *MIST1* loci are hypermethylated in PDAC cells (B. Marshall, B. Jakubison & S. F. Konieczny, unpublished data), our next direction will be to activate endogenous *MIST1*/*PTF1a* gene expression by reversing the methylated status of these loci. Technologies such as modified CRISPR targeting (e.g., dCas9‐SunTag; CRISPR/Cas9 TGA) to demethylate CpG islands at the *MIST1* and *PTF1a* regulatory regions could have a significant impact on PDAC and progenitor and acinar cell transcription networks, possibly leading to reduced aggressiveness of the disease (Liao *et al*., 2017; Morita *et al*., 2016). Additional studies investigating expression of other acinar transcription factors such as GATA6 and NR5a2 will also be of importance as each is essential to the acinar differentiation program and each suppresses KRAS‐mediated ADM/preneoplastic transformation (von Figura *et al*., 2014; Flandez *et al*., 2014; Martinelli *et al*., 2016). These and other efforts will provide additional insights regarding the early stages of PDAC development and facilitate the advancement of novel strategies to successfully treat the disease.

## 5 Conclusions

In this study, we asked whether induced expression of acinar transcription factors in PDAC tumor cells could partially activate the acinar gene program and at the same time reduce tumor cell properties. Both MIST and PTF1a induced target genes that are normally associated with differentiated acinar cells. Additionally, induction of PTF1a in PDAC influenced cancer‐associated properties, leading to decreases in cell proliferation, CSC numbers, and repression of ABC efflux transporters resulting in heightened sensitivity to gemcitabine. These results suggest that manipulation of the PDAC transcriptome offers promising opportunities to develop novel therapeutic intervention strategies for this deadly disease.

## Conflict of interest

The authors declare no conflict of interest.

## Author contributions

BLJ, PGS, SM, and YY designed, performed, and analyzed experiments; HG and YL assisted with bioinformatics; KS and PI‐A provided cellular reagents and advice; and BLJ, PGS, and SFK wrote the manuscript.

## Supporting information


**Doc. S1.** Differentially expressed gene promoter *de novo* motif analysis.

**doc** **. S2.** IPA cancer‐associated molecules.

**Fig. S1.** Generation of the Tet‐inducible cell lines.
**Fig. S2.** Comparison of Tet‐MIST1‐ and Tet‐PTF1a‐induced targets with human pancreas samples.
**Fig. S3.** BxPC‐3 and KPC cells engineered to express Tet‐MIST1 and Tet‐PTF1a.
**Fig. S4.** PTF1a and MIST1 differentially expressed promoter motif enrichment and ChIP‐Seq intersect.
**Fig. S5.** KEGG pathway analysis of the Tet‐PTF1a and Tet‐MIST1 RNA‐Seq.
**Table S1.** Antibodies.
**Table S2.** Mouse and human RT‐qPCR primers.
