# Supplementary material for: Induced PTF1a expression in pancreatic ductal adenocarcinoma cells activates acinar gene networks, reduces tumorigenic properties, and sensitizes cells to gemcitabine treatment
Source: Mol Oncol. 2018 May 21;12(7):1104–24. doi: 10.1002/1878-0261.12314 (PMC6026875; doi:10.1002/1878-0261.12314)
Supplement: Supplementary file 1 — Doc. S1. Differentially expressed gene promoter de novo motif analysis. [file MOL2-12-1104-s001.pdf]

Promoter Motifs

Tet-PTF1a Induced DEG Promoter Motifs

Rank	Motif	P-value	% of Targets	% of Background	Best Match/Details
1	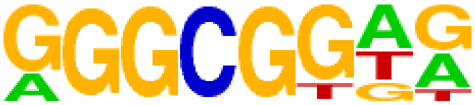	1e-127	81.35%	61.69%	POL003.1_GC-box/Jaspar(0.845)
2	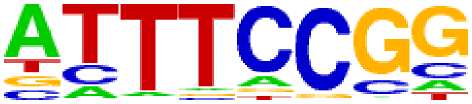	1e-80	64.70%	47.90%	Elk4(ETS)/Hela-Elk4-ChIP-Seq(GSE31477)/Homer(0.941)
3	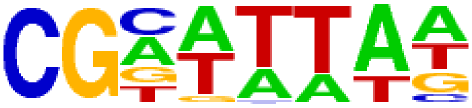	1e-60	60.54%	46.04%	PH0126.1_Obox6/Jaspar(0.679)
4	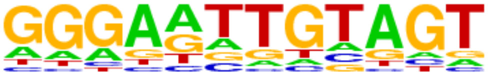	1e-58	7.37%	2.12%	GFY(?)/Promoter/Homer(0.985)
5	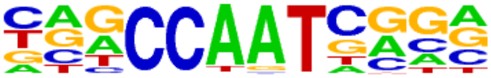	1e-53	31.40%	19.84%	NFY(CCAAT)/Promoter/Homer(0.957)
6	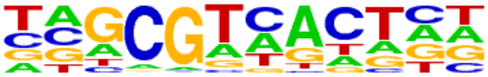	1e-46	64.45%	51.85%	Atf1/MA0604.1/Jaspar(0.768)
7	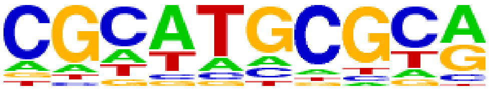	1e-44	59.86%	47.46%	NRF(NRF)/Promoter/Homer(0.912)
8	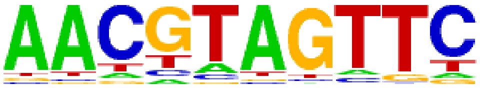	1e-29	18.40%	11.46%	PB0161.1_Rxra_2/Jaspar(0.605)
9	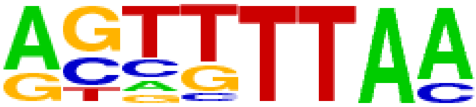	1e-25	59.61%	50.26%	PB0109.1_Bbx_2/Jaspar(0.741)
10	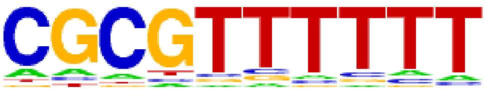	1e-22	33.05%	25.17%	PB0192.1_Tcfap2e_2/Jaspar(0.669)

Tet-PTF1a Repressed DEG Promoter Motifs

Rank	Motif	P-value	% of Targets	% of Background	Best Match/Details
1	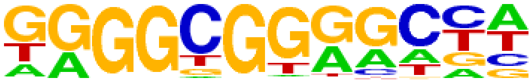	1e-92	63.06%	45.35%	POL003.1_GC-box/Jaspar(0.939)
2	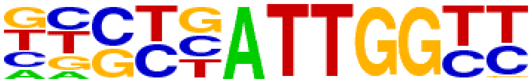	1e-49	19.41%	10.60%	NFY(CCAAT)/Promoter/ Homer(0.960)
3	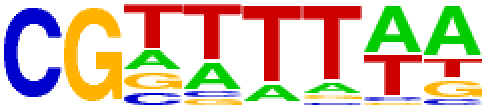	1e-48	61.94%	49.18%	PH0044.1_Homez/ Jaspar(0.713)
4	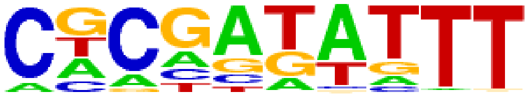	1e-28	40.66%	31.51%	PH0023.1_Dlx4/ Jaspar(0.643)
5	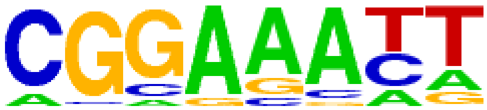	1e-25	38.00%	29.47%	Elk4(ETS)/Hela-Elk4-ChIP-Seq(GSE31477)/ Homer(0.857)
6	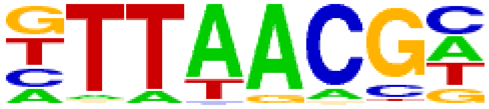	1e-24	50.11%	41.20%	MYBL1/MA0776.1/ Jaspar(0.816)
7	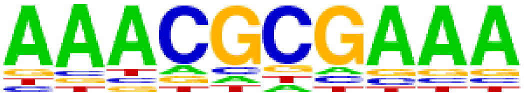	1e-20	51.23%	43.06%	IRF2/MA0051.1/ Jaspar(0.668)
8	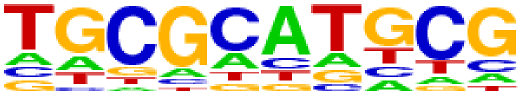	1e-19	23.71%	17.36%	NRF(NRF)/Promoter/ Homer(0.906)
9	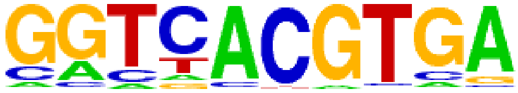	1e-17	11.11%	7.02%	E-box(bHLH)/Promoter/ Homer(0.946)
10	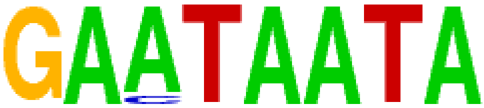	1e-16	87.53%	82.15%	MF0010.1_Homeobox_class /Jaspar(0.754)

TET-MIST1 INDUCED DEG PROMOTER MOTIFS

Rank	Motif	P-value	% of Targets	% of Background	Best Match/Details
1	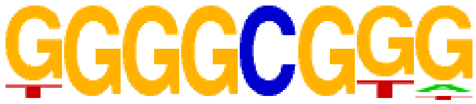	1e-32	74.33%	49.38%	PB0110.1_Bcl6b_2/ Jaspar(0.904)
2	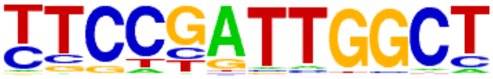	1e-25	25.85%	10.06%	NFY(CCAAT)/Promoter/ Homer(0.884)
3	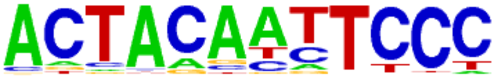	1e-16	10.95%	3.12%	GFY(?)/Promoter/ Homer(0.976)
4	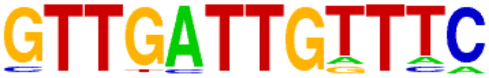	1e-12	1.62%	0.03%	Dux/MA0611.1/ Jaspar(0.774)
5 *	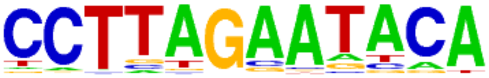	1e-11	9.16%	3.04%	PB0194.1_Zbtb12_2/ Jaspar(0.651)
6 *	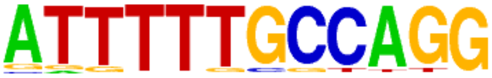	1e-10	2.33%	0.16%	PB0145.1_Mafb_2/ Jaspar(0.689)
7 *	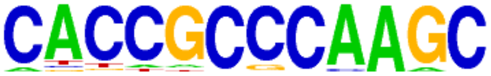	1e-10	3.23%	0.38%	EGR3/MA0732.1/ Jaspar(0.715)
8 *	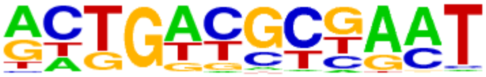	1e-10	2.15%	0.13%	CREB1/MA0018.2/ Jaspar(0.644)
9 *	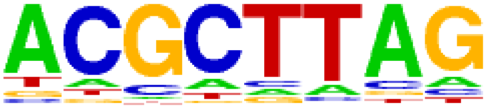	1e-10	42.01%	28.77%	Nr2e3/MA0164.1/ Jaspar(0.673)
10 *	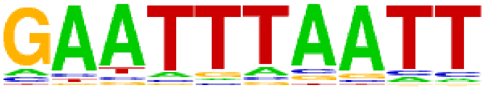	1e-10	17.59%	8.69%	PROP1/MA0715.1/ Jaspar(0.778)

**Possible False Positive* (printed in italics)

TET-MIST1 REPRESSED DEG PROMOTER MOTIFS

Rank	Motif	P-value	% of Targets	% of Background	Best Match/Details
1	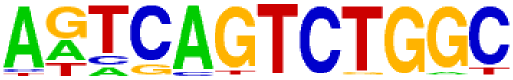	1e-14	4.71%	0.19%	Smad2(MAD)/ES-SMAD2-ChIP-Seq(GSE29422)/ Homer(0.753)
2	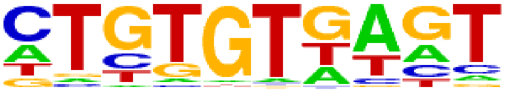	1e-12	11.45%	2.41%	PB0119.1_Foxa2_2/ Jaspar(0.691)
3	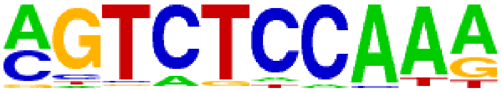	1e-12	10.44%	2.02%	PB0160.1_Rfxdc2_2/ Jaspar(0.740)
4 *	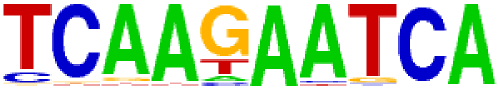	1e-11	7.74%	1.18%	Pdx1(Homeobox)/Islet-Pdx1-ChIP-Seq(SRA008281)/ Homer(0.727)
5 *	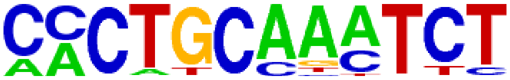	1e-11	5.72%	0.57%	PB0091.1_Zbtb3_1/ Jaspar(0.674)
6 *	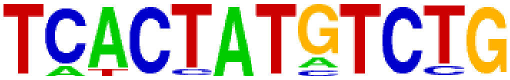	1e-11	2.69%	0.05%	Smad2(MAD)/ES-SMAD2-ChIP-Seq(GSE29422)/ Homer(0.589)
7 *	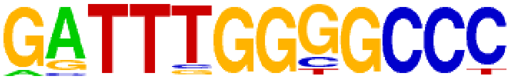	1e-11	4.04%	0.23%	ZNF692(Zf)/HEK293-ZNF692.GFP-ChIP-Seq(GSE58341)/ Homer(0.708)
8 *	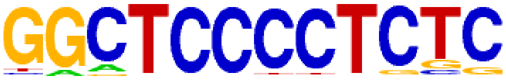	1e-11	7.41%	1.14%	MZF1/MA0056.1/ Jaspar(0.667)
9 *	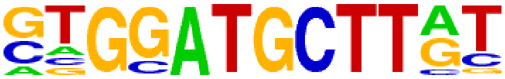	1e-10	4.04%	0.24%	Nr2e3/MA0164.1/ Jaspar(0.660)
10 *	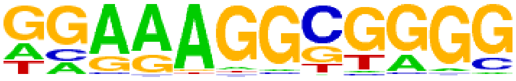	1e-10	8.42%	1.62%	SP2/MA0516.1/ Jaspar(0.723)

**Possible False Positive*
