# Supplementary material for: Induced PTF1a expression in pancreatic ductal adenocarcinoma cells activates acinar gene networks, reduces tumorigenic properties, and sensitizes cells to gemcitabine treatment
Source: Mol Oncol. 2018 May 21;12(7):1104–24. doi: 10.1002/1878-0261.12314 (PMC6026875; doi:10.1002/1878-0261.12314)
Supplement: Supplementary file 3 — Fig. S1. Generation of the Tet‐inducible cell lines. Fig. S2. Comparison of Tet‐MIST1‐ and Tet‐PTF1a‐induced targets with human pancreas samples. Fig. S3. BxPC‐3 and KPC cells engineered to express Tet‐MIST1 and Tet‐PTF1a. Fig. S4. PTF1a and MIST1 differentially expressed promoter motif enrichment and ChIP‐Seq intersect. Fig. S5. KEGG pathway analysis of the Tet‐PTF1a and Tet‐MIST1 RNA‐Seq. Table S1. Antibodies. Table S2. Mouse and human RT‐qPCR primers. [file MOL2-12-1104-s003.pdf]

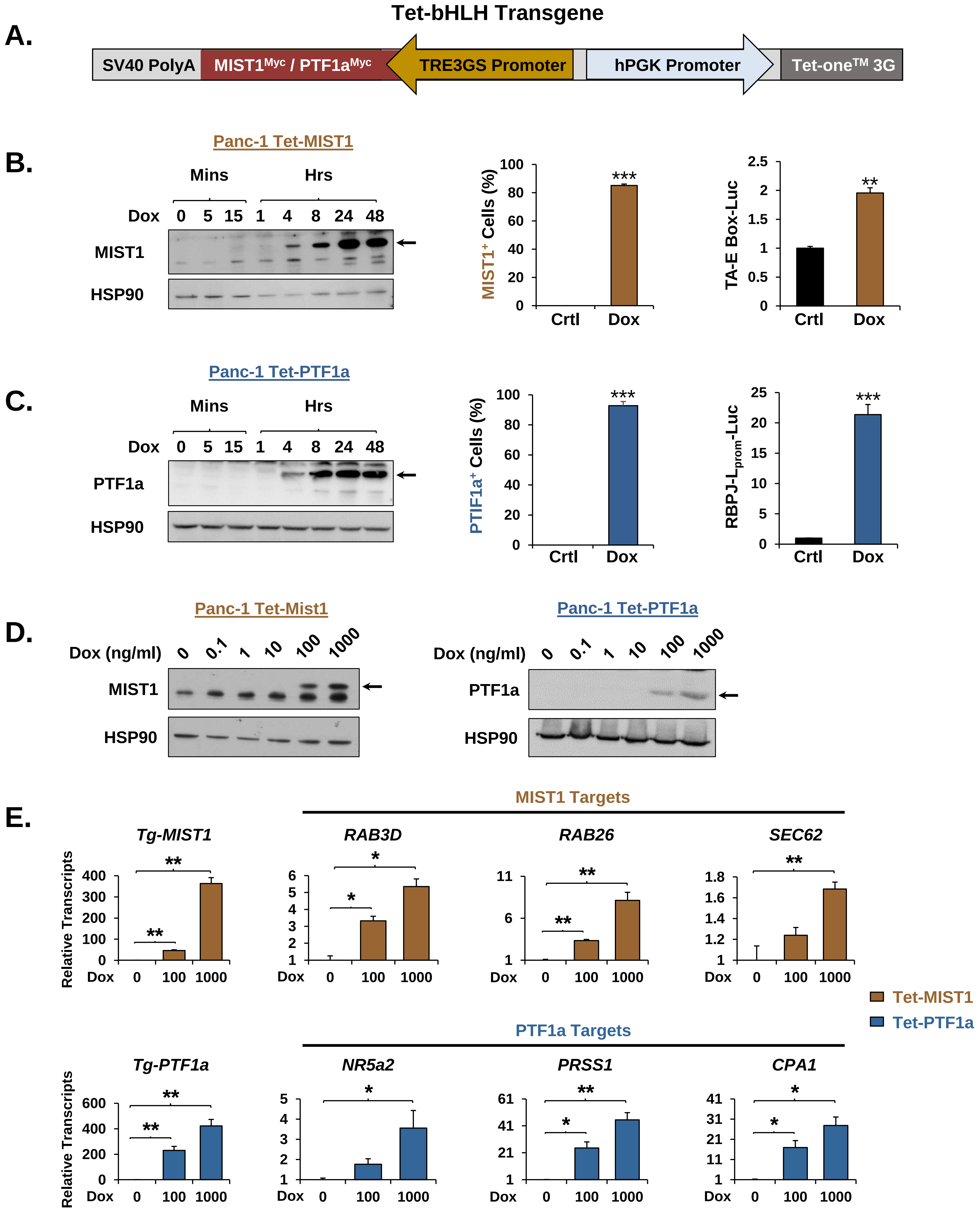

FIGURE S1: *Generation of the Tet-inducible cell lines.* **(A)** Schematic of Tet-inducible *MIST1*^{Myc} and *PTF1a*^{Myc} transgenes. **(B-C)** Immunoblot, IHC and luciferase assays of MIST1 and PTF1a expression upon doxycycline treatment. Both MIST1 and PTF1a are detected at 4 hours post-doxycycline with maximal expression reached at 24 hours. **(D)** Tet-MIST1 and Tet-PTF1a cells were treated with the indicated dox concentrations for 72 hours. Induced MIST1 and PTF1a is detectable with 100 ng/ml dox with maximum levels of expression obtained with 1 µg/ml dox. **(E)** RT-qPCR of MIST1 and PTF1a target genes from Tet-MIST1 and Tet-PTF1a cells treated with the indicated dox concentrations. * P ≤ 0.05, ** P ≤ 0.01, *** P ≤ 0.001.

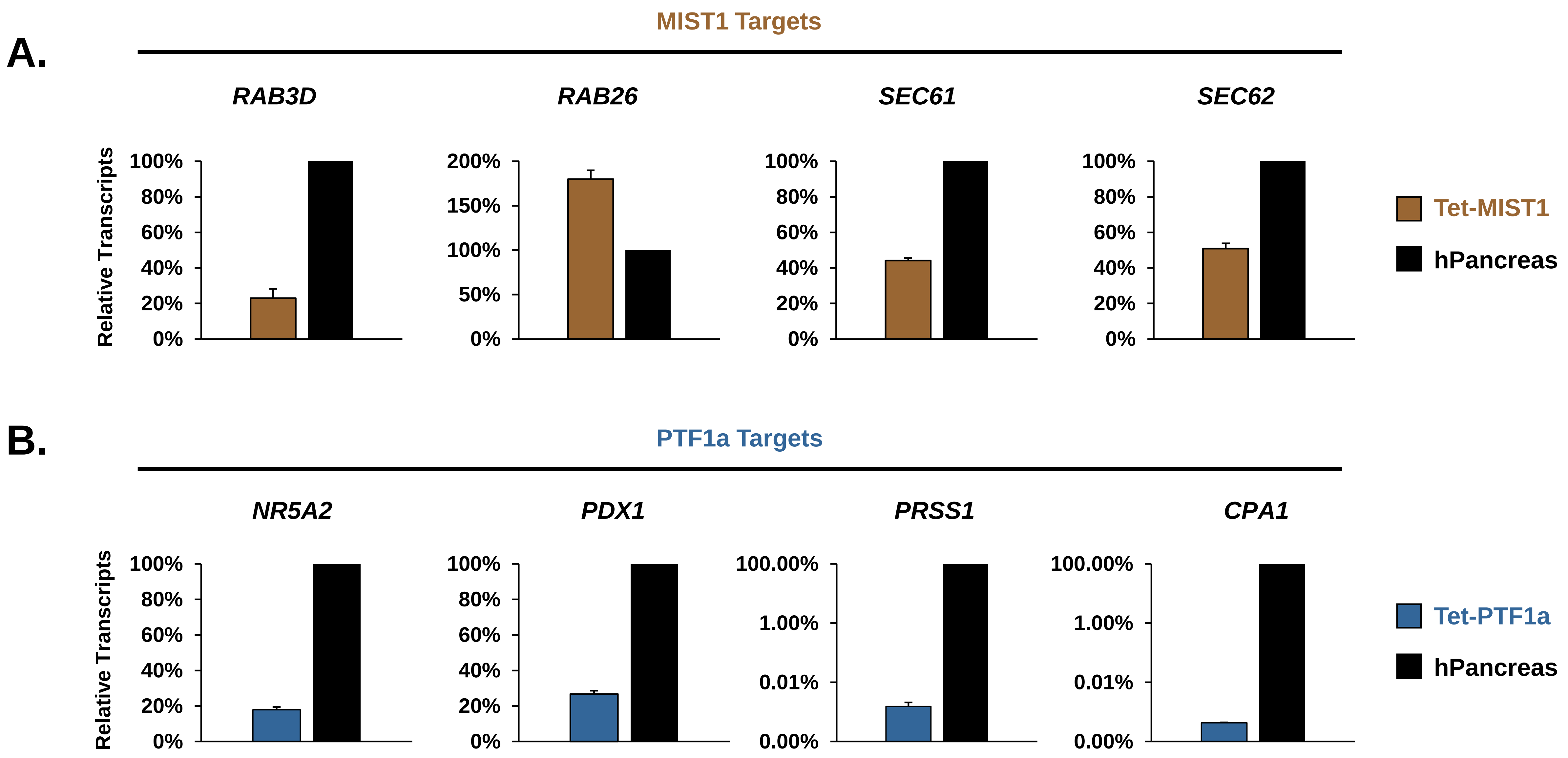

Figure S2: *Comparison of Tet-MIST1 and Tet-PTF1a induced targets with human pancreas samples.* **(A)** Tet-MIST1 and **(B)** Tet-PTF1a cell RNA was subjected to RT-qPCR and compared to transcript levels in a normal human pancreas sample. Note that only one human RNA sample was tested in this experiment so no statistics are presented.

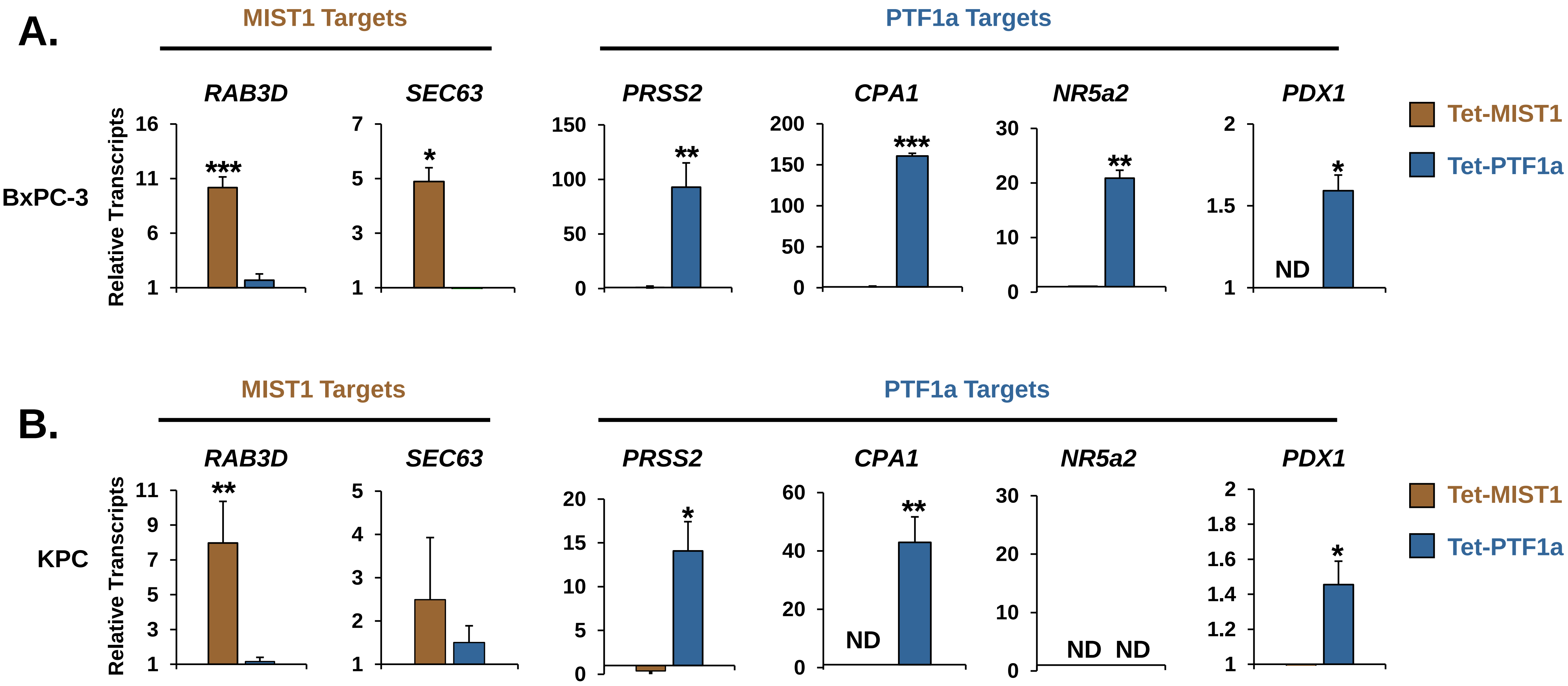

FIGURE S3: *BxPC-3 and KPC cells engineered to express Tet-MIST1 and Tet-PTF1a.* Tet-MIST1 and Tet-PTF1a human BxPC-3 and mouse KPC PDAC lines were generated and gene expression was analyzed by RT-qPCR. **(A)** MIST1 and PTF1a target gene expression in BxPC-3 cells. **(B)** MIST1 and PTF1a target gene expression in KPC cells. In both cases MIST1 and PTF1a are able to induce appropriate gene expression profiles in the PDAC lines.
* P ≤ 0.05, ** P ≤ 0.01, *** P ≤ 0.001. ND - not detected.

A.

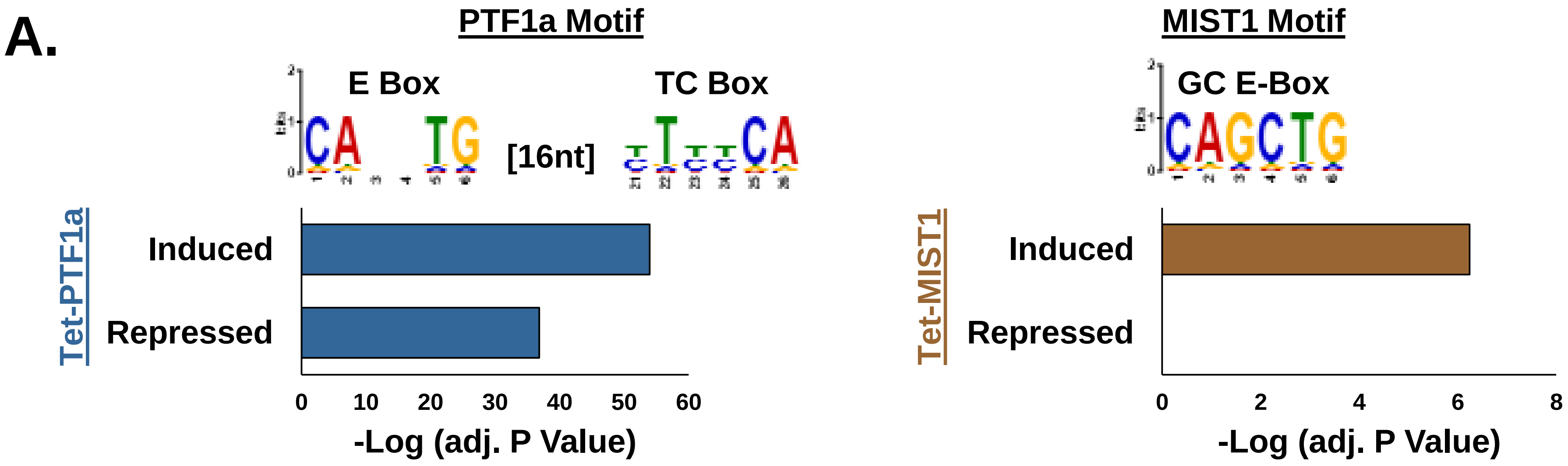

B.

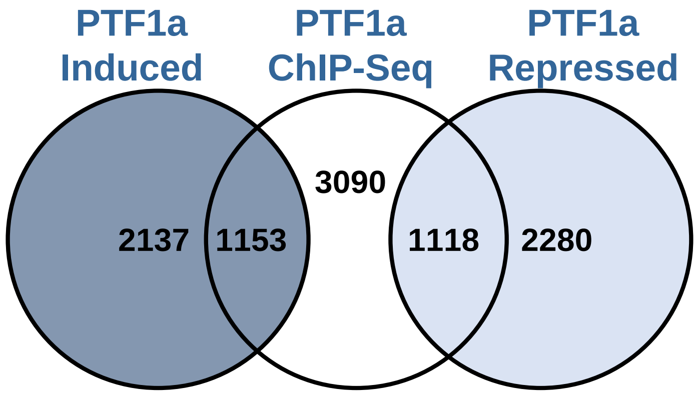

Category	Tet-Ptf1a DEG Overlap with Ptf1a ChIP-Seq
Pancreas TF Network	MIST1, NR5A2, PDX1
ER Stress Control	ATF6, EIF2B1, DNAJC3, HYOU1, HSPB1, XBP1
Unfolded Protein Binding	CALR, DNAJB1
ER-Associated Degredation	EDEM3, PSMD6, SEL1L, UCHL5, NFE2L2
Translocon	RRBP1, SVIP, TRAM1
Disulfide Bonds	ALG3, QSOX2
Digestive Enzymes	PRSS1
Notch Signaling	DLL1, HIF1A, HES1, MAML3, PSEN2, RBPJ

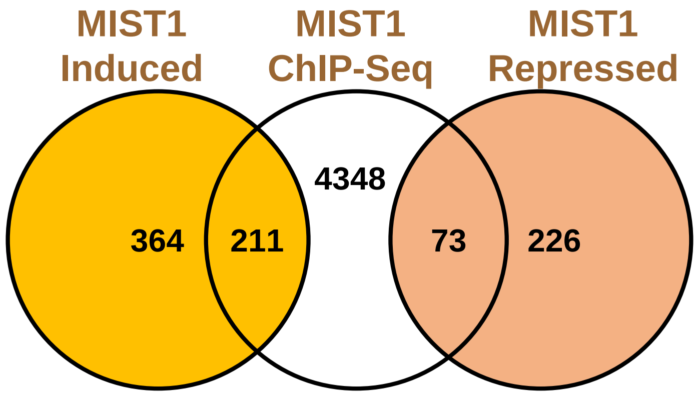

Category	Tet-Mist1 DEG Overlap with Mist1 ChIP-Seq
Protein Export	SEC61B, SEC63
Secretion	RAB3D, RAB26, RAB27A, DNAJC1
Regulation of Translation	PAIP2B
Protein Ubiquitination	UBA5
Metabolism	BCKDK
Transmembrane Transport	ABCB6

FIGURE S4: *PTF1a and MIST1 differentially expressed promoter motif enrichment and ChIP-Seq intersect.* **(A)** AME (MEME-Suite) was used to examine the enrichment of PTF1a or MIST1 motifs in the promoters of differentially expressed genes from the respective RNA-Seq experiments. In addition to the motif enrichment results displayed, PTF1a motifs with one helical turn between the E box and TC Box and MIST1 TA E box motifs were also included in the analysis, but were not enriched in any dataset and are therefore not represented in the figure. **(B)** Intersect between the differentially expressed genes from the RNA-Seq and genes with ChIP-Seq peaks from previously published mouse studies. Highlighted genes induced in the RNA-Seq and identified in the ChIP-Seq are shown in the accompanying tables. The majority of the targets listed here have been previously identified and reported in past studies.

A.

Tet-PTF1a Induced Genes			
ID	Name	#Gene	FDR
hsa04141	Protein processing in endoplasmic reticulum - Homo sapiens (human)	66	4.92E-11
hsa05169	Epstein-Barr virus infection - Homo sapiens (human)	56	3.10E-03
hsa04390	Hippo signaling pathway - Homo sapiens (human)	45	3.10E-03
hsa04330	Notch signaling pathway - Homo sapiens (human)	19	5.46E-03
hsa03008	Ribosome biogenesis in eukaryotes - Homo sapiens (human)	33	5.46E-03
hsa03040	Spliceosome - Homo sapiens (human)	39	5.67E-03
hsa04144	Endocytosis - Homo sapiens (human)	64	8.56E-03
hsa04392	Hippo signaling pathway -multiple species - Homo sapiens (human)	13	8.56E-03
hsa04360	Axon guidance - Homo sapiens (human)	47	8.56E-03
hsa04722	Neurotrophin signaling pathway - Homo sapiens (human)	35	8.56E-03

B.

Tet-PTF1a Repressed Genes			
ID	Name	#Gene	FDR
hsa01100	Metabolic pathways - Homo sapiens (human)	324	5.62E-12
hsa01200	Carbon metabolism - Homo sapiens (human)	54	4.79E-11
hsa05205	Proteoglycans in cancer - Homo sapiens (human)	73	6.33E-08
hsa01212	Fatty acid metabolism - Homo sapiens (human)	25	5.62E-06
hsa00280	Valine, leucine and isoleucine degradation - Homo sapiens (human)	24	1.87E-05
hsa04066	HIF-1 signaling pathway - Homo sapiens (human)	40	1.87E-05
hsa01230	Biosynthesis of amino acids - Homo sapiens (human)	31	6.90E-05
hsa05016	Huntington's disease - Homo sapiens (human)	61	7.54E-05
hsa05200	Pathways in cancer - Homo sapiens (human)	107	8.72E-05
hsa04015	Rap1 signaling pathway - Homo sapiens (human)	64	1.91E-04

C.

Tet-MIST1 Induced Genes			
ID	Name	#Gene	FDR
hsa01100	Metabolic pathways - Homo sapiens (human)	62	2.03E-04
hsa00280	Valine, leucine and isoleucine degradation - Homo sapiens (human)	9	6.55E-04
hsa00640	Propanoate metabolism - Homo sapiens (human)	6	1.84E-02
hsa03060	Protein export - Homo sapiens (human)	5	2.35E-02
hsa00760	Nicotinate and nicotinamide metabolism - Homo sapiens (human)	5	5.82E-02
hsa00510	N-Glycan biosynthesis - Homo sapiens (human)	6	9.70E-02
hsa00630	Glyoxylate and dicarboxylate metabolism - Homo sapiens (human)	4	2.64E-01
hsa00790	Folate biosynthesis - Homo sapiens (human)	3	2.64E-01
hsa00750	Vitamin B6 metabolism - Homo sapiens (human)	2	3.42E-01
hsa01200	Carbon metabolism - Homo sapiens (human)	8	3.58E-01

D.

Tet-MIST1 Repressed Genes			
ID	Name	#Gene	FDR
hsa05146	Amoebiasis - Homo sapiens (human)	7	1.31E-01
hsa05217	Basal cell carcinoma - Homo sapiens (human)	4	8.12E-01
hsa05200	Pathways in cancer - Homo sapiens (human)	12	8.12E-01
hsa04360	Axon guidance - Homo sapiens (human)	7	8.12E-01
hsa00604	Glycosphingolipid biosynthesis - ganglio series - Homo sapiens (human)	2	8.12E-01
hsa04390	Hippo signaling pathway - Homo sapiens (human)	6	8.12E-01
hsa05205	Proteoglycans in cancer - Homo sapiens (human)	7	8.12E-01
hsa04512	ECM-receptor interaction - Homo sapiens (human)	4	8.12E-01
hsa04340	Hedgehog signaling pathway - Homo sapiens (human)	3	8.12E-01
hsa04015	Rap1 signaling pathway - Homo sapiens (human)	7	8.12E-01

FIGURE S5: *KEGG pathway analysis of the Tet-PTF1a and Tet-MIST1 RNA-Seq.* The top 10 reported KEGG pathways from independent analysis of the induced and repressed differentially expressed genes are shown from **(A,B)** the Tet-PTF1a or **(C,D)** the Tet-MIST1 RNA-Seq.

Supplemental Table 1: *Antibodies.*

Antibodies					
**Target**	**Dilution**	**Purpose**	**Species**	**Source**	**Catalog Number**
α-MIST1	1:100 ; 1:1000	IF; WB	Rabbit	In-house	-
α-PTF1a	1:50 ; 1:500	IF; WB	Rabbit	Wright Lab	-
α-CPA1	1:100	IF; WB	Rabbit	Santa Cruz	131901
α-PRSS2	1:100; 1:000	IF; WB	Mouse	Sigma	sab1400226
α-PRSS2	1:100	IF	Rabbit	Abcam	ab223064
α-MYC	1:500 ; 1:500	IF; WB	Mouse	In-house	9E10
α-HSP90	1:5000	WB	Rabbit	Santa Cruz	sc-7947
α-Sox9	1:3000	IF; WB	Rabbit	Millipore	ab5535
α-K19	1:500	IF; WB	Rat	Dev. Studies	Troma3
α-Amylase	1:250	IF	Rabbit	Abcam	ab21156
α-S6	1:2000	WB	Mouse	Santa Cruz	Sc-74459
α-CD24 FITC	1:100	Flow	Mouse	BD Bioscience	560992
α-CD44 PE Cy5	1:100	Flow	Rat	BD Bioscience	553135
α-EPCAM	1:500	WB	Rat	BD Bioscience	740389
α-Caspase 3	1:500	WB	Rabbit	Cell Signaling	9662
α-PDX1	1:1000	WB	Rabbit	Santa Cruz	H-140
α-Cleaved Caspase 3	1:500	WB	Rabbit	Abcam	ab2302

SUPPLEMENTAL TABLE 2: *Mouse and human RT-qPCR primers.*

qRT-PCR Primers		
**Transcript**	**Primer 1 Sequence**	**Primer 2 Sequence**
**Transgenes**		
Mist1 (tg)	AGCATTACCATCACCAGCAG	CCCTCTCTGAAGCTGTGGAT
Ptf1a (tg)	AGGTTATCATCTGCCATCGAG	GACACAAACTCAAAGGGTGGT
**Human**		
huMist1	CCAGCACTACCAGCAGCA	AGGACTGGGCGCTAGGTG
huPtf1a	AGAAGGTCATCATCTGCCATC	GTCCTTTAGGACCCTTTCCTG
Rab26	CGATTCAAGGATGGTGCTTT	CCGGTAGTAGGCATGGGTAA
Rab3d	GGGGAACAAGTGTGACCTG	CAGGGACTCGTTCATCTTCTC
Sec61b	GGGGGCTCCGTAACTTTCTA	TGTGTAGAATCGCCACATCC
Sec62	GGATCCAGGAAGTTGGTGAA	CCTCTCCTTTCTTGGCCTTT
Sec63	GATCAGAATGCCGAGCAAAT	TAAGAACAATGCCCATCCTG
Perk	GCCTTGGATCCTGAAAATCA	GTCTTGGTCCCACTGGAAGA
Dnajc1	CGGAGACCTGGAGTTGTTTG	CTGCCTTCGTTCATCATCCT
Prss1	TCTGTCCCCTACCAGGTGTC	TCTTGGCTGCATTGATGAAC
Prss2	GTTGCAGCTGCTGTTGCTGCC	TGTCATTGTCCAGAGTCCGGC
Cpa1	TGTCTTTGGCAAGGAGGACT	ACGTCCTCGATCATGGTCTC
Cpa2	AGTGGGTTACACAAGCTACG	AGAGGCTTCCAGATACCTTG
Cela3b	GGGGCCGTCTCTATACCAAT	CAGAAACAAAGCTGGTCACA
Amy2a	CAGACCTTGGTGGGAAAGATA	GGTACTGCTTGTTCCTGCACT
Cd44	TCCAACACCTCCCAGTATGACA	GGCAGGTCTGTGACTGATGTACA
Cd24	ATGTGGCAAGGAAAAACAGG	CTCCATTCCACAATCCCATC
Cd44v6	GGAACAGTGGTTTGGCAACAG	TTGGGTGTTTGGCGATATCC
Aldh1a3	GCAACCTGGAGGTCAAGTTC	CCTCTGGAAGGCAACCTGT
Abcg2	GGGTGACTCATCCCAACATT	AAGCCATTGGTGTTTCCTTG
Abcb1	TGATTGCATTTGGAGGACAA	CCAGAAGGCCAGAGCATAAG
Abcc1	GCTTTCAGAACACGGTCCTC	AGAGAGTTGGGCTGACCAGA
Abcc2	CTGCCACTTTGTTTTGAGCA	GTGAGTACAAGGGCCAGCTC
Abcc3	CTCCAAGTTCTGGGACTCCA	TGGGAGAGGATGATGTAGCC
Abcc4	TTCTTCTGGTGGCTCAATCC	GCTTCTGTGCGTCATTCTCA
Abcc5	TGCCGCTATAAAGGCTTGTT	AGTGCTGGTTCTCTCCCTCA
Abcc6	GGAACCAGACAGAGCCTGAA	GGAACCAGACAGAGCCTGAA
Ent1	CTGCCTGAGCCTGAACTAGG	CTGGTGACTGGTTGTCATGG
Cnt1	CTTTGCCTTTCAGGTTCTGC	CAAGTAGGGCCGGATCAGTA
Nr5a2	GGGTACCATTATGGGCTCCT	GGCCCAAACTTATTCCTTCC
Pdx1	AGCTGCCTTTCCCATGGAT	GGAACTCCTTCTCCAGCTCT
Gata6	AGTTTTCCGGCAGAGCAGTA	GTCAGTCAAGGCCATCCAC
18s	GATATGCTCATGTGGTGTTG	AATCTTCTTCAGTCGCTCCA
Epcam	AATGTGTGTGCGTGGGA	TTCAAGATTGGTAAAGCCAGT
Aldh2	AGACCCTAGCTCTGCTCTCG	GGGAATGTTTTCCTGCTGAC
**Mouse**		
msMIST1	TGGTGGCTAAAGCTACGTGT	CATAGCTCCAGGCTGGTTTT
msPTF1a	AGGTTATCATCTGCCATCGAG	GACACAAACTCAAAGGGTGGT
msAmy	CAGAGACATGGTGACAAGGTG	ATCGTTAAAGTCCCAAGCAGA
msSox9	CACGGAACAGACTCACATCTC	CCTCTCGCTTCAGATCAACTT
msK19	CCTCCCGAGATTACAACCACT	AGGCGTGTTCTGTCTCAAACT
msRplp0	AGAAACTGCTGCCTCACATCC	CAATGGTGCCTCTGGAGATT
msRab3d	AGTGTGACCTGGAAGACGAAC	CCAGGGATTCATTCATCTTGT
msSec63	GCGGAGCAAATTCGATTAAA	TGTTGCTCCAGGATCCAAAT
msPRSS2	TCTGTGGAGGTTCCCTCATC	ACTCTGGCATTGAGGGTCAC
msCPA1	ACCTTTGAAGGTCGTCCCAT	TGTTGTCAAGAATGGCCGTG
msNR5a2	CCCAAACGTGAGGAACAACT	TATCGCCACACACAGGACAT
msPDX1	ATGAAATCCACCAAAGCTCAC	GGTCAAGTTCAACATCACTGC
